# Post-symptomatic NLRP3 inhibition rescues cognitive impairment and mitigates amyloid and tau driven neurodegeneration

**DOI:** 10.1038/s44400-025-00011-5

**Published:** 2025-05-06

**Authors:** Anick Auger, Rania Faidi, Alexis D. Rickman, Carolina Pena Martinez, Austin Fajfer, Jeremy Carling, Addison Hilyard, Mubashshir Ali, Ryosuke Ono, Connor Cleveland, Ria Seliniotakis, Nhi Truong, Amandine Chefson, Marianne Raymond, Marie-Anne Germain, Michael A. Crackower, Bradlee L. Heckmann

**Affiliations:** 1 Ventus Therapeutics, Inc., 4800 Rue Levy, Montreal, QC, H4R 2P7 Canada; 2 USF Health Byrd Alzheimer’s Center and Neuroscience Institute, Department of Molecular Medicine Morsani College of Medicine, Tampa, FL 33613 USA; 3 Ventus Therapeutics U.S., Inc., 100 Beaver Street, Suite 201, Waltham, MA 02453 USA

**Keywords:** Neurodegeneration, Neurodegenerative diseases, Drug discovery, Immunology, Neuroscience

## Abstract

Emerging evidence has established neuroinflammation as a primary driver of progressive neuronal loss observed across neurodegenerative diseases (NDDs). The NLRP3 inflammasome is a cytosolic immunoprotective danger sensing complex, which when aberrantly activated drives neuroinflammation, propagates amyloid deposition, and neurodegeneration. Herein, we report the therapeutic benefit of NLRP3 inflammasome inhibition in Alzheimer’s disease (AD), using a novel and selective brain-penetrant small molecule NLRP3 inhibitor, VEN-02XX, which we profiled in the 5XFAD/Rubicon KO AD model. We demonstrate for the first time that targeting NLRP3, post-symptomatic establishment, rescues cognitive deficits, mitigates neuronal loss, and is sufficient to significantly reduce reactive microgliosis, neuroinflammation and tau pathology. Our data further suggest that pharmacological inhibition of NLRP3, after disease onset, has the potential to reduce cortical and hippocampal amyloid burden. Together, these results highlight the potential for NLRP3 inhibition as a symptomatic and disease modifying therapeutic target for AD pathology and more broadly NDDs.

## Introduction

Neurodegenerative diseases (NDDs) are a family of neurological disorders primarily characterized by the progressive degeneration of neurons within the central and peripheral nervous system. NDDs include Alzheimer’s disease (AD), Parkinson’s disease (PD) and amyotrophic lateral sclerosis (ALS), diseases characterized by a diverse array of cognitive and motor symptoms. Despite diverse clinical presentation, NDDs share common pathological hallmarks. For instance, they are all characterized by the cellular deposition of misfolded protein aggregates, specifically β-amyloid (Aβ) and tau in AD, α-synuclein in PD and TAR DNA-binding protein (TDP-43) in ALS^1^.

Over the last decade, therapeutic intervention has focused primarily on targeting these protein aggregates. However, limited clinical success has been achieved via this route. In AD, for example, Aβ targeted antibody approaches have recently demonstrated limited clinical efficacy in terms of slowing disease progression, but with an enhanced risk of adverse events^2^. As such, the unmet needs of AD and other NDDs remain substantial.

Emerging evidence has established a role for neuroinflammation as a primary driver of the progressive neuronal cell death observed in neurodegenerative diseases^2,3^. In the pathogenic brain, microglia are activated in response to various signals including α-synuclein, Aβ, tau pathology, neuronal injury, and feedback from other activated glia, leading to robust inflammatory cytokine production. Dysregulation of microglial homeostasis and chronic activation or reactive microgliosis propagate and accelerate further deposition of protein aggregates leading to exacerbated neurodegeneration^4–6^. Interestingly, these signaling events and impaired regulation of neuroinflammation are now well-defined across the majority of NDDs^7–10^.

While activated microglia produce a variety of proinflammatory cytokines, interleukin (IL)-1β has been shown to be highly correlated to neurodegenerative pathology and is significantly elevated in both post-mortem human AD and PD brain samples and murine models of AD and PD^7–13^. Microglial-derived IL-1β activates neuronal death through multiple mechanisms including ER-stress pathway activation, increased pro-apoptotic Bax expression and enhanced cytochrome C release from neuronal mitochondria, activation of p53 signaling, and indirect mechanisms including increased production of reactive oxygen species (ROS)^14–18^. In addition to promoting neurodegeneration, IL-1β drives neuronal dysfunction and signaling impairment through alteration of synaptic structure, plasticity, and altered NMDA/AMPA receptor dynamics^19–21^. Cumulatively, this combination of death induction and neuronal signaling impairment are clearly observed in neurodegenerative diseases like AD, PD and ALS.

IL-1β is produced by the NLRP3 inflammasome, a multimeric protein complex comprised of NLRP3 (NOD-, LRR- and pyrin domain-containing protein 3), ASC (apoptosis-associated speck-like protein containing a caspase recruitment domain), and pro-caspase 1^22,23^. Upon activation, NLRP3 self-oligomerizes, binds the adapter protein ASC, subsequently activating caspase-1 which proteolytically processes IL-1β and IL-18 cytokines to generate the mature forms and induce the highly inflammatory form of cell death known as pyroptosis through the activation of Gasdermin D (GSDMD)^24^. Since cytokines are primary mediators of inflammatory processes within the central nervous system (CNS), NLRP3 activation in brain cells, and specifically microglia, directly contributes to neuroinflammation. Canonical activation of the pathway is best characterized in response to pathogen-associated molecular patterns (PAMPs) and danger-associated molecular patterns (DAMPs) following recognition by NLRP3^22^ and NLRP3 is emerging as a perturbation detector for cellular stress and cell membrane damage^24^. In the context of neurodegenerative diseases, multiple stimuli are known to activate NLRP3 including α-synuclein^25^, Aβ^26^, tau^27^, and reactive oxygen species (ROS)^28^.

Recent studies have shown the loss of conjugation of ATG8/LC3 to single membranes (CASM) sensitizes cells towards inflammasome activation and IL-1β production. In the context of NDDs, CASM-deficiency driven by the ablation of the key regulator Rubicon, results in exacerbated neuroinflammation and neurodegeneration^29–33^. The 5XFAD/Rubicon KO model is based on the standard 5XFAD mouse, which drives AD pathology via humanized mutations in APP and PS1 leading to Aβ plaque formation as observed in human patients. The 5XFAD/Rubicon KO mouse model recapitulates multiple markers of human neurodegenerative disease and specifically AD pathology, including robust Aβ deposition, select tau pathology, region specific neurodegeneration, memory and cognitive impairment, and reactive microgliosis, neuroinflammation, and IL-1β architecture that mimics human disease^29–33^. The increased and accelerated pathophysiology of the 5XFAD/Rubicon KO model is a result of reduced microglial receptor recycling and enhanced neuronal production of Aβ, leading to increased Aβ deposition and suppression of Aβ clearance and a direct effect of the loss of single membrane LC-3 lipidation on regulating inflammatory pathway activation including NLRP3 signaling^29,30,32,34^. However, the broader contribution of inflammasome activation and IL-1β production as a key driver of pathology downstream of CASM-deficiency remains elusive.

Genetic modulation of the NLRP3 inflammasome and inhibition of IL-1β signaling via ablation of either NLRP3 or caspase-1 provides robust neuronal protection, preserves spatial memory, and mitigates other key disease sequelae in multiple murine models of AD^35,36^. However, pharmacological inhibition of the NLRP3 inflammasome in the context of the CNS has proven challenging due to multiple confounding factors including lack of compound specificity and blood brain barrier (BBB) permeability. In the present study, we utilized a novel, highly selective, CNS-penetrant and orally bioavailable NLRP3 inhibitor, VEN-02XX, as a mouse tool molecule. To determine the therapeutic capacity of VEN-02XX in neurodegenerative diseases, and the contribution of inflammasome signaling to the observed AD pathology downstream of CASM-deficiency, the 5XFAD/Rubicon knockout mice were treated following symptomatic and neuropathological establishment.

As shown herein, therapeutic intervention using VEN-02XX significantly reduced microgliosis and neuroinflammation, limited neurodegeneration, and dramatically improved memory and cognition in a dose-dependent manner. Although not statistically significant, our data further suggest that NLRP3 inhibition could reduce amyloid burden. Together, these results further validate the robust potential of the NLRP3 inflammasome and IL-1β signaling as a therapeutic target with symptomatic and disease modifying potential in NDDs and establish for the first time the robust contribution of inflammasome activation as a driving factor downstream of CASM-deficiency.

## Results

### VEN-02XX is brain penetrant and potently and selectively inhibits the NLRP3 inflammasome

**Fig. 1:**
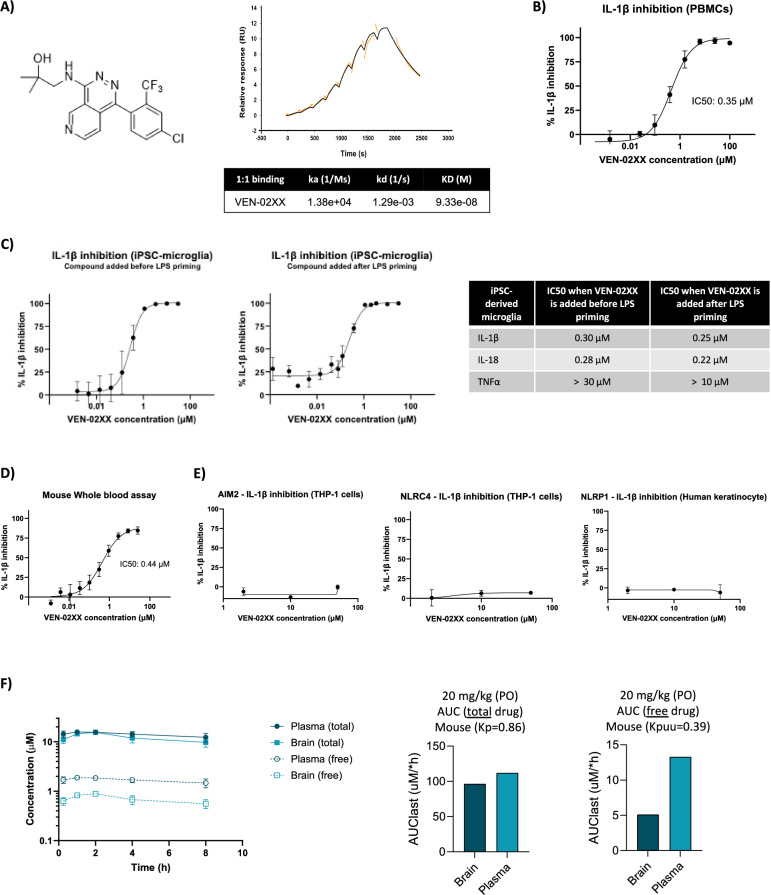
VEN-02XX is a selective and brain penetrant NLRP3 inhibitor. **A** VEN-02XX chemical structure and SPR analysis of VEN-02XX interaction with human NLRP3. **B**–**E** The half maximal inhibitory concentration (IC50) value is the geometric mean of at least 2 independent experiments (PBMCs *n* = 3; iPSC-derived microglia *n* = 5; activity against other nod-like receptors (NLRs) *n* = 2; mouse whole blood (*n* = 3). **F** VEN-02XX total and unbound brain-to-plasma partition coefficient, Kp (0.86) and Kp,uu, (0.39) brain (mouse). Both Kp and Kp,uu were calculated using the area under the curve (AUClast) of a pharmacokinetic study in WT mice following single oral dosing with VEN-02XX at 20 mg/kg.

**Fig. 2:**
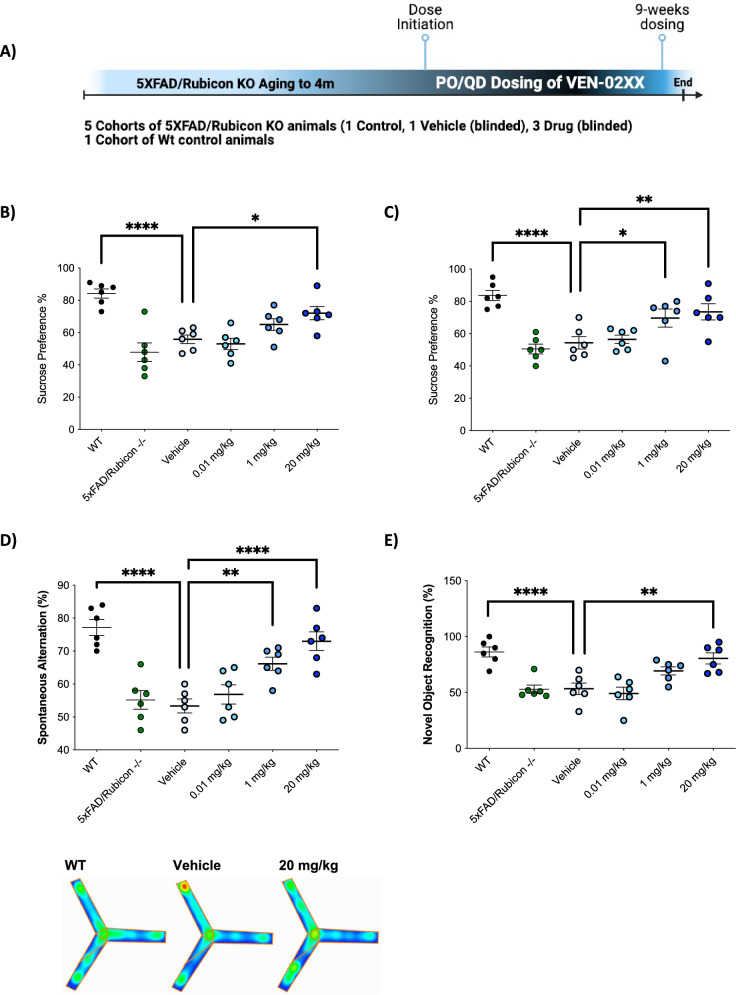
Inhibition of NLRP3 using VEN-02XX reverses cognitive impairment and restores memory in a preclinical model of AD. **A** VEN-02XX treatment schematic in 5XFAD/Rubicon KO mice with established AD pathology. Sucrose preference test performed at **B** 6-weeks and **C** 9-weeks post-dose initiation. **D** Spatial working and short-term memory evaluation using Y-maze and **E** novel object recognition. *p < 0.05, **p < 0.01, ****p < 0.00001.

Using unique structural biology and protein engineering capabilities, Ventus Therapeutics has generated multiple chemical series of NLRP3 inhibitors. From this effort, Ventus Therapeutics discovered and is developing a CNS penetrant NLRP3 inhibitor, VENT-02, for the treatment of NDDs, and recently completed a Phase I healthy volunteer trial. After characterization of the various chemical series, VEN-02XX was identified as being an optimal compound for animal studies with a potent and selective profile along with demonstrated CNS-penetration in mice. Specifically, VEN-02XX binds to NLRP3 with high affinity (*K*_D_ = 93.3 nM) with a residence time of 775 s (1/kd) demonstrating that it is a specific and reversible NLRP3 inhibitor (Fig. 1A). VENT-02XX binding to NLRP3 leads to NLRP3 inhibition in various cellular systems with sub-micromolar potency including peripheral blood mononuclear cell (PBMC) (Fig. 2B), iPSC-derived microglia (Fig. 1C) and whole blood (Fig. 1D). VEN-02XX specificity for NLRP3 is demonstrated by the lack of TNFα inhibition in cellular assay (Fig. 1C) as well as by its inability to block IL-1β release downstream of other inflammasomes like absent in melanoma 2 (AIM2), NOD-like-receptor containing a Pyrin domain 1 (NLRP1) and the NLR-family CARD domain-containing protein 4 (NLRC4) (Fig. 1E). In addition, with an unbound brain-to-plasma partition coefficient, Kp,uu brain of 0.39 in mice, VEN-02XX can penetrate the CNS to inhibit the NLRP3 protein that is localized to the brain cells and therefore is able to directly modulate neuroinflammation (Fig. 1F). With these properties, VEN-02XX was identified to be the optimal NLRP3 inhibitor to interrogate for the first time the therapeutic role of NLRP3 in a mouse model of neurodegeneration. Pharmacokinetic studies in WT mice following 7 days of oral dosing once daily with VEN-02XX at 20 mg/kg demonstrated that this dose is sufficient to maintain a 50% inhibitory activity of NLRP3 throughout the dosing schedule (Supplementary Fig. 1).

### Inhibition of NLRP3 using VEN-02XX restores memory and cognition

The overt symptomatic hallmark of AD in human patients is the loss of hippocampal-dependent learning and memory^37^. Neuroinflammation driven by sustained NLRP3 inflammasome activation and IL-1β signaling has become a well-defined pathological mechanism promoting neurodegeneration. The 5XFAD/Rubicon KO mouse model used herein recapitulates multiple facets of human neurodegenerative diseases including robust Aβ deposition, tau phosphorylation, neuronal loss, reactive microgliosis, neuroinflammation including NLRP3 activation and IL-1β production, cumulatively leading to memory and learning impairment^14,26,35^.

To evaluate the efficacy of NLRP3 inhibition in NDDs after disease onset, we treated 5XFAD/Rubicon KO mice with varying doses of VEN-02XX or matched vehicle via oral administration once daily (PO/QD) for a total of 9 weeks after presentation of disease pathology. 5XFAD/Rubicon KO mice display robust moderate to severe AD pathology by 4 months of age^29–33^. To confirm behavioral impairment as a baseline for initiating treatment and to randomize animals into dosing cohorts, we performed a sucrose preference test (SPT) to test for anhedonia (inability to sense pleasure). Anhedonia is commonly reported in AD patients and has been shown previously in the model^32^. At 4 months of age, the 5XFAD/Rubicon KO mice exhibited a significant reduction in sucrose preference compared to wild type littermates with no changes in water consumption (58.9 vs. 84, *p* < 0.0001) (Supplementary Fig. 2A, B), confirming anhedonia and consistent with previous results^32^. Following confirmation of symptomatic deficits, VEN-02XX or vehicle dosing was initiated in the 4-month-old 5XFAD/Rubicon KO animals (Fig. 2A).

As early as 6-weeks post-treatment with VEN-02XX, a trending reversal in the pre-dosing anhedonia was observed with the lower dose treatments, and significant improvement was seen in the 20 mg/kg dose group (Fig. 2B). By 9-weeks, a dose-dependent response was clearly exhibited with significant improvements in both the 1 mg/kg and 20 mg/kg groups with no changes in water consumption (Fig. 2C and Supplementary Fig. 2C, D).

To evaluate hippocampal-dependent learning and memory more robustly, we performed endpoint (9-week) analyses for both spatial and working memory using a Y-maze and novel object recognition. A striking dose-dependent improvement was observed for animals on VEN-02XX intervention compared to vehicle treated animals with respect to both spontaneous alternation (Fig. 2D) and novel object recognition (Fig. 2E) with the 20 mg/kg group displaying restoration of learning and memory to near wild type control levels for most animals.

Taken together, these data demonstrate for the first time that NLRP3 inhibition, after disease onset and using a brain penetrant compound (VEN-02XX) leads to robust symptomatic efficacy with specific improvements in learning and memory in the context of AD pathology. Moreover, VEN-02XX reversed behavioral impairment as early as 6-weeks of dosing in a dose-dependent manner.

### VEN-02XX reverses microgliosis and mitigates neuroinflammation

Reactive microgliosis and neuroinflammation are primary drivers of neuronal impairment and neuronal cell death in both human AD and the 5XFAD/Rubicon KO mouse model^32^. Unrestricted inflammation both in the hippocampus and brain in general, lead to a host of sequelae including neurodegeneration driven by pro-inflammatory cytokines including IL-1β and TNFα amongst others^32^. The primary source of pro-inflammatory cytokines in NDDs including AD is through localized production by microglial cells. Microglia are the innate immune cell of the CNS and are similar to their peripheral macrophage counterparts with respect to both function and signaling^38,39^. In the AD brain, microglia become activated in response to Aβ, dying cells, injured neurons, and a variety of other stimuli and progress from homeostatic to reactive states^40–43^. Moreover, inflammatory microglia in the context of NDDs are often further delineated into categories of disease associated microglia (DAMs) which are characterized by significant morphological and genetic changes^44^. Reactive ameboid shaped microglia are a characteristic of human post-mortem NDD brains and are likewise observed in the 5XFAD/Rubicon KO mouse.

**Fig. 3:**
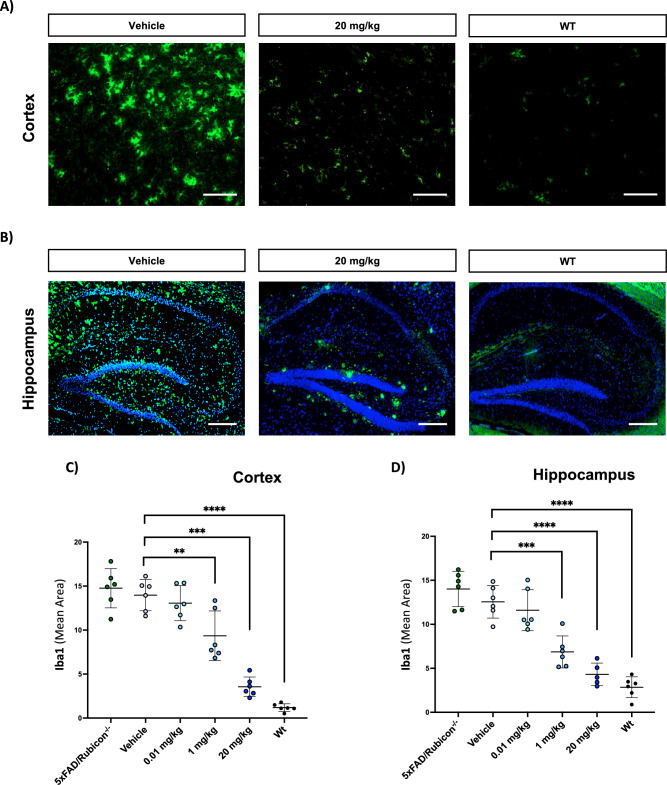
VEN-02XX treatment ameliorates pathological microgliosis. Evaluation of microglial activation via Iba1 staining in cortex (**A**) and hippocampus (**B**), Iba1-green, DAPI-blue. Scale bars, 50 um for (**A**), 100 um for (**B**). Quantification of cortical (**C**) and hippocampal (**D**) Iba1 mean area. Each point represents the average area measures from 16-fields per image of two slides/animal for all study animals per group (*n* = 6). Data are presented as mean ± SEM. **p < 0.01, ***p < 0.0001, ****p < 0.00001.

**Fig. 4:**
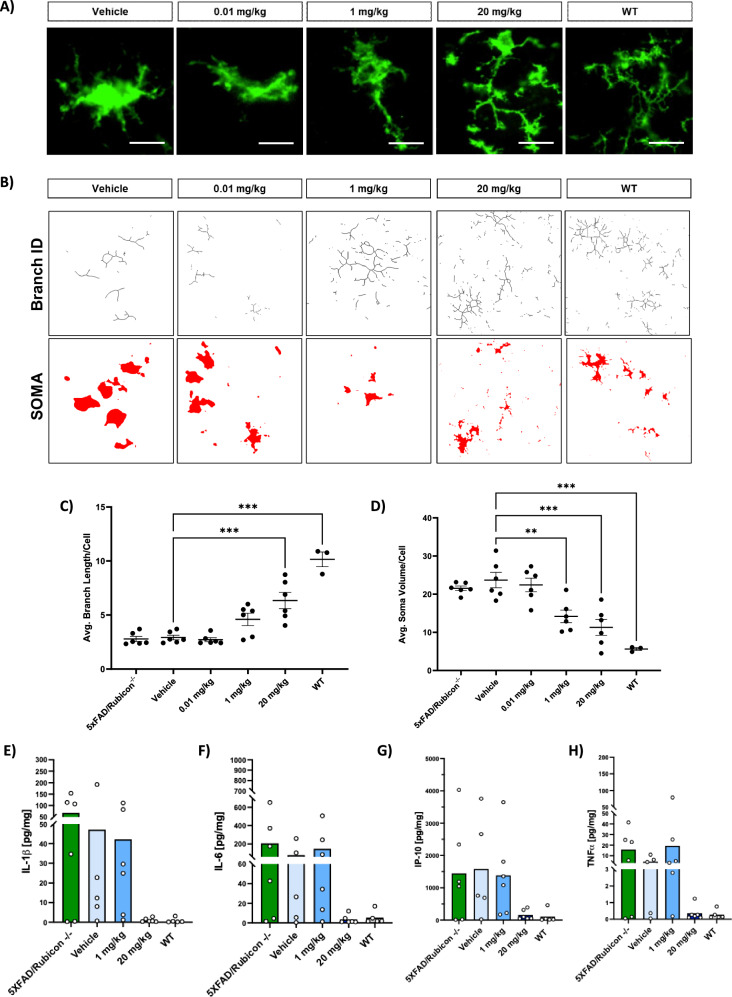
VEN-02XX treatment restores homeostatic microglial architecture and reduces neuroinflammation. **A** Gross evaluation of microglial morphology via Iba1 staining. Scale bars, 10 um. **B** Representative 3DMorph skeletal and soma analyses of microglial morphology. **C**, **D** Quantification of microglial branch length and soma volume respectively (Each point represents the average measured values for a minimum 400 cells/animal, *n* = 3 for WT and *n* = 6 for all other groups). **E**–**H** Quantification (pg/mL) of hippocampal IL-1β (total), IL-6, IP-10, and TNFα in 5XFAD Rubicon knockout (−/−) mice receiving no treatment (green bars; *n* = 6), vehicle (light blue bars; *n* = 5), and 1 mg/kg (medium blue bars; *n* = 6) or 20 mg/kg VEN-02XX (dark blue bars; *n* = 6). Wild type (WT) mice receiving no treatment (*n* = 5) are shown in gray bars. Data are presented as mean ± SEM. *p < 0.05, **p < 0.01, ***p < 0.0001.

Based on the mechanism of action of blocking NLRP3 activation, we had hypothesized that VEN-02XX treatment would result in suppression of IL-1β signaling and lead to a reduction in microglial activation. Therefore, we evaluated brains following the 9-week treatment intervention from the VEN-02XX treated groups compared to vehicle controls and naïve 5XFAD/Rubicon KO age-matched animals. We found that VEN-02XX reversed the reactive microgliosis observed in the 5XFAD/Rubicon KO model. VEN-02XX treatment reduced the cumulative signal of the key microglial activation marker, Iba1, in both the cortex and hippocampus in a dose-dependent and statistically significant manner (Fig. 3A–D and Supplementary Fig. 3). Gross morphological analysis revealed a transition from the disease-associated active ameboid morphology towards a homeostatic ramified morphology upon exposure to VEN-02XX (Fig. 4A). These findings were further supported by skeletal analysis of the branch/projections and the overall soma volume (Fig. 4B). VEN-02XX 20 mg/kg treated animals had significant increases in microglial branch length (Fig. 4C), with commensurate decreases in soma volume in a dose-dependent and statistically significant manner (Fig. 4D).

Similar to patients with other NDDs, AD patients as well as animal models of AD exhibit increased levels of IL-1β, IL-6, IP-10, and TNFα, particularly in the hippocampus where efferent cortical and subcortical projections affected by AD originate^45,46^. To quantify the impact of VEN-02XX on neuroinflammation in 5XFAD/Rubicon KO mice, several inflammatory cytokines and chemokine with known roles in AD pathology were measured^47^. A notable reduction in all measured inflammatory markers was observed in the 20 mg/kg VEN-02XX group, showing levels comparable to those observed in the WT mice, suggesting an anti-inflammatory effect mediated by NLRP3 blockade despite the lack of statistical significance (Fig. 4E–H). Interestingly, the 5XFAD/Rubicon KO animals presented with a more dampened inflammatory profile within the cortex compared to hippocampus and as such the effects of VEN-02XX were similarly reduced (Supplementary Fig. 4A–D). However, a similar trend of reduced levels in the 20 mg/kg VEN-02XX group was observed (Supplementary Fig. 4A–D).

Consistent with our hypothesis, VEN-02XX intervention shifted the microglial phenotype of the 5XFAD/Rubicon KO model from a hyper-activated ameboid state to a more homeostatic ramified state with reductions in major pro-inflammatory cytokines including an almost complete ablation of IL-1β within the hippocampus. To our knowledge, this is the first demonstration that NLRP3 pharmacological inhibition is directly impacting microglia morphology in vivo.

### NLRP3 inhibition using VEN-02XX altered Aβ burden and microglial-plaque association

Classical hallmarks of AD include Aβ-containing plaques and hyperphosphorylated tau with associated neuroinflammation and neurodegeneration^47^. We examined the induction of Aβ deposition and microglial recruitment to Aβ plaques which is a common feature of both human AD and the 5XFAD/Rubicon KO model, which has enhanced plaque deposition compared to the traditional 5XFAD mouse with robust plaque deposition established by 3–4 months of age^29–33^.

**Fig. 5:**
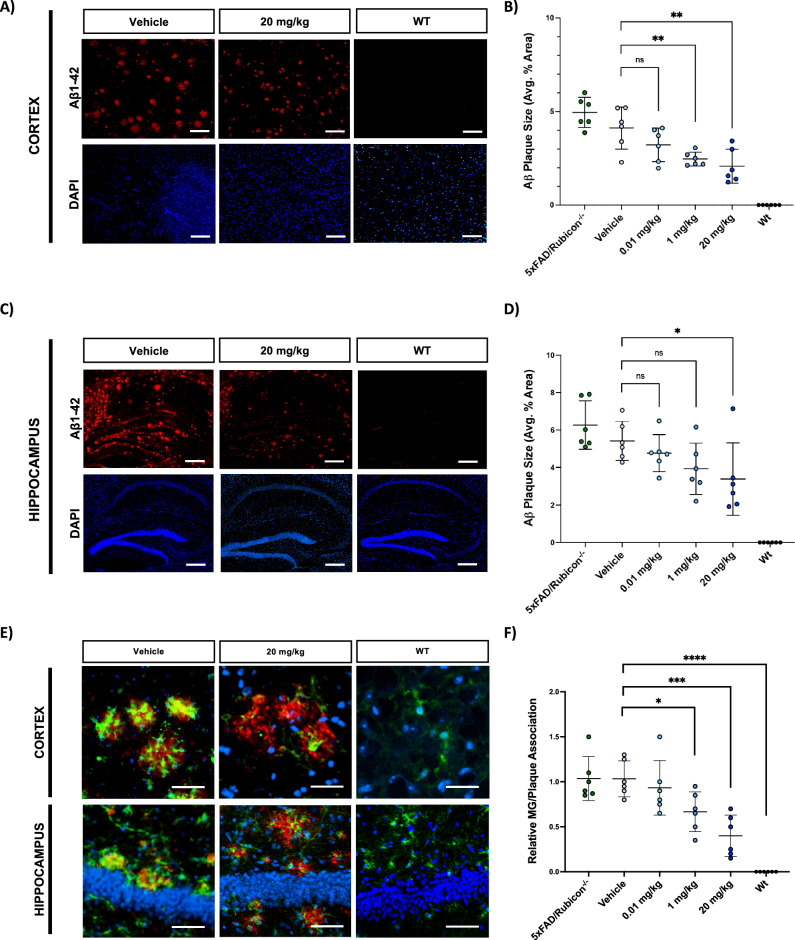
VEN-02XX treatment reduces amyloid burden and disease-associated plaque localization. **A** Representative immunofluorescence imaging for amyloid plaques (red) and DAPI (blue) in cortex. Scale bars, 10 um. **B** Quantification of cortical amyloid plaque area. **C** Representative immunofluorescence imaging for amyloid plaques (red) and DAPI (blue) in hippocampus. Scale bars, 100 um. **D** Quantification of amyloid plaque area in hippocampus. **E**, **F** Representative images and quantification evaluating microglial (Iba1-green) association with amyloid plaques (red), DAPI (blue). Scale bars, 20 um. MG/Plaque association was quantified as the co-localization of Iba1 with amyloid and represented as relative to 5xFAD/Rubicon^−/−^. Data are presented as mean ± SEM. **p < 0.01, ***p < 0.0001, ****p < 0.00001.

In addition to reducing microglial activation, VEN-02XX therapeutic treatment resulted in a significant reduction in Aβ plaque area/size in both the cortex and hippocampus in a dose-dependent manner, especially at higher doses (Fig. 5A–D). A trending reduction in Aβ plaque burden (cumulative plaque #) was likewise observed in both the cortex and hippocampus, although these data did not reach statistical significance (Fig. 5A, C and Supplementary Fig. 3A–D). This dose-response trend in Aβ plaque reduction in both size and putatively overall plaque counts following therapeutic dosing with VEN-02XX, further supports a putative role for NLRP3 in driving the Aβ plaque burden in AD. Specifically, published reports have demonstrated a link between NLRP3 activation and Aβ deposition in animal models of AD, particularly in driving the seeding and propagation^48^. In light of our therapeutic dosing with VEN-02XX in the 5XFAD/Rubicon KO mice with established AD pathology^32^, it is plausible that blocking NLRP3 reduced Aβ deposition and spreading throughout the 9-week dosing period. Preventing further deposition and propagation of Aβ could explain the consistent trend of Aβ plaque size and number reduction in both the cortex and the hippocampus with increasing doses of VEN-02XX (Fig. 5A–D and Supplementary Fig. 3). It is also possible that these dose-dependent trends are a direct consequence of restoring microglial function through NLRP3 inhibition which would lead to Aβ clearance and plaque size reductions in addition to enhanced clearance of pre-plaque forms including highly neurotoxic monomeric and oligomeric species.

Moreover, VEN-02XX treatment caused a shift in the microglial architecture characterized by a robust disassociation of the microglia from the Aβ plaques (Fig. 5E, F). Plaque-associated microglia are typically observed in post-mortem brains from AD patients, and it is currently held that plaque-associated microglia are a surrogate for disease progression and severity^49–51^.

Cumulatively, these data support the suppression of microglial activation through inhibition of NLRP3 using a brain penetrant inhibitor (VEN-02XX), leading to a reversal in the disease-associated neuroinflammation which ultimately is contributing to alleviating Aβ pathology.

### NLRP3 inhibition with VEN-02XX dampens tau pathology and preserves neuronal populations

In addition to Aβ deposition, the second classical hallmark of AD pathology is the hyperphosphorylation and aggregation of tau. Together with neuroimmune activation, these events precede neurodegeneration^52^. In human AD, tau is phosphorylated at a variety of sites leading to the formation of neurofibrillary tangles^53,54^. In non-humanized tau models of AD pathology in mice, tau hyperphosphorylation and aggregation is often not present. However, the 5XFAD/Rubicon KO mouse displays both cortical and hippocampal phosphorylation of endogenous murine tau starting by 4 months of age, although no neurofibrillary tangling has been observed, likely due to the intrinsic biophysical differences in human versus murine tau protein^55^. This timeline is accelerated when compared to the canonical 5XFAD mouse model, which also features tau phosphorylation and in certain instances aggregation typically between 6–8 months of age compared to ~4 months for the 5XFAD/Rubicon KO model^56,57^.

**Fig. 6:**
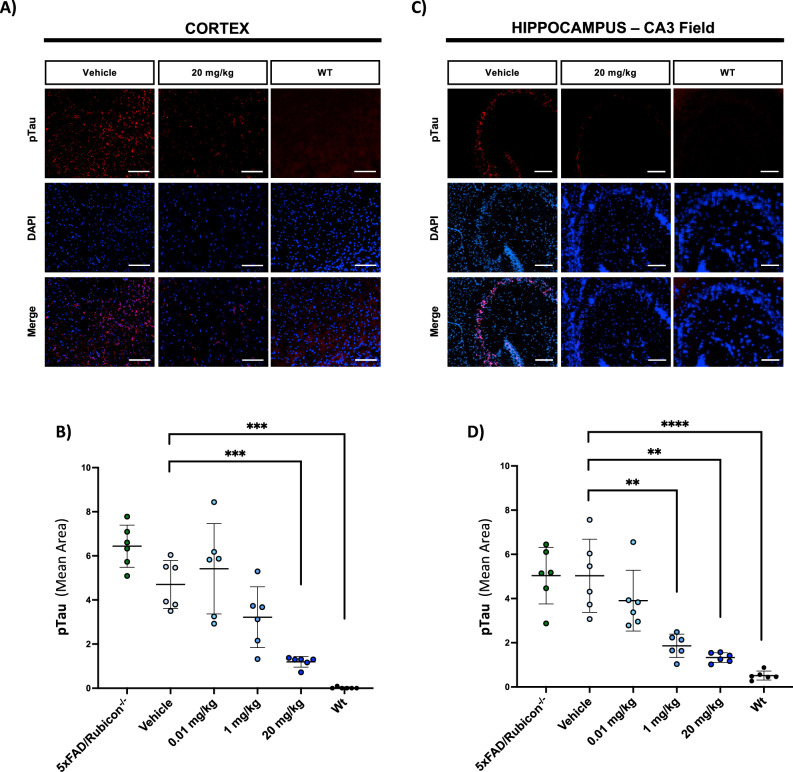
VEN-02XX treatment limits endogenous tau phosphorylation. Representative immunofluorescence imaging for pTau S396 (red) and DAPI (blue) in cortex (**A**) and hippocampus (**C**). Scale bars, 50 um for (**A**), 100 um for (**C**). **B**, **D** Quantification of pTau S396 mean area in cortex and hippocampus respectively. Data are presented as mean ± SEM. **p < 0.01, ***p < 0.0001.

We evaluated the phosphorylation status of tau in the 5XFAD/Rubicon KO mice in response to NLRP3 inhibition by therapeutic treatment with VEN-02XX. Overall, a dose-dependent and statistically significant reduction in phosphorylated tau (pTau) was observed in mice treated with VEN-02XX with an almost complete suppression in pTau signal in the 20 mg/kg treated group in both the cortex (Fig. 6A, B and Supplementary Fig. 5A) and the CA3 region of the hippocampus (Fig. 6C, D and Supplementary Fig. 5B). Together, these data suggest that reduced neuroinflammation through inhibition of NLRP3 by VEN-02XX suppresses tau pathology, further limiting neuronal death.

### VEN-02XX is neuroprotective and reduces markers of neuronal death in the brain

**Fig. 7:**
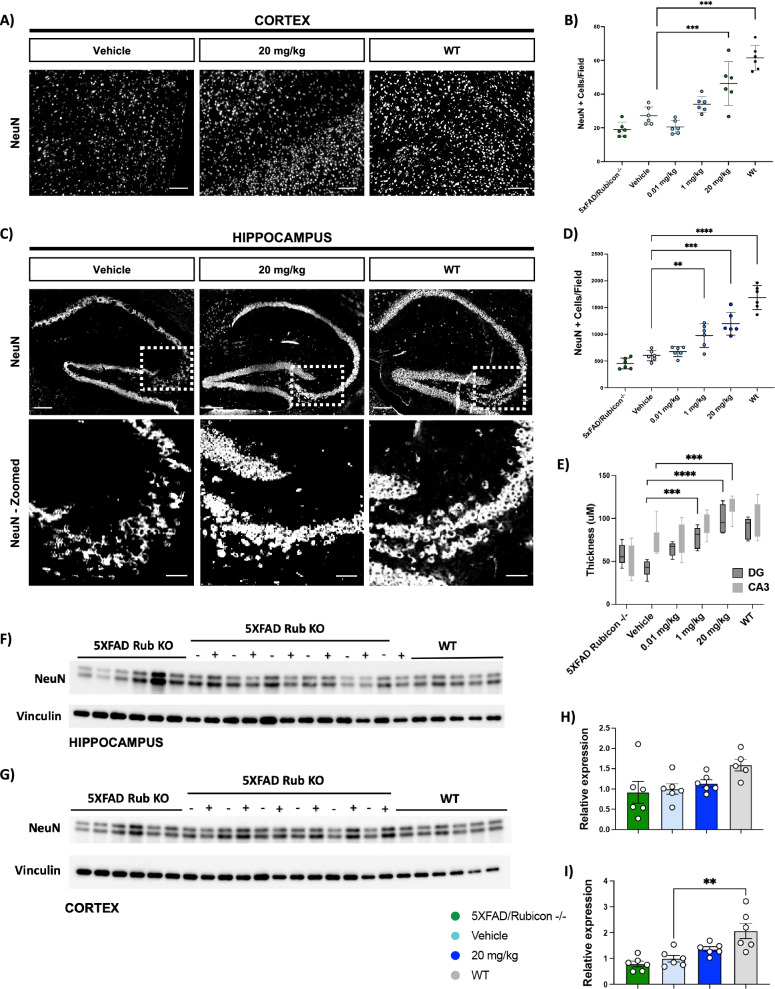
VEN-02XX treatment restricts neurodegeneration and improves neuronal architecture. **A** Representative immunofluorescence imaging of cortical neurons (NeuN—white). Scale bars, 100 um. **B** Quantification of NeuN + cell count in cortex. **C** Representative immunofluorescence imaging of hippocampal neurons (NeuN—white). Scale bars, 100 um for *top*, 10 um for *bottom*. **D** Quantification of NeuN + cell count in hippocampus. For cortical and hippocampal NeuN quantification, each point represents the average NeuN+ cell counts from 16-fields per image of two slides/animal for all study animals per group (*n* = 6). **E** Measurement of the CA3 and dentate gyrus thickness measured in uM, on NeuN stained sections. **F** Immunoblot analysis for NeuN expression in hippocampal lysates; (−) animals refer to vehicle dosed 5XFAD/Rubicon KO mice and (+) animals refer to 20 mg/kg VEN-02XX dosed 5XFAD/Rubicon KO mice. **G** Immunoblot analysis for NeuN expression in cortical lysates; (−) animals refer to vehicle dosed 5XFAD/Rubicon KO mice and (+) animals refer to 20 mg/kg VEN-02XX dosed 5XFAD/Rubicon KO mice. **H** Quantification of band intensity for NeuN normalized to vinculin for hippocampal samples in **F** (same protein gel along with MAP2). **I** Quantification of band intensity for NeuN normalized to vinculin for cortical samples in **G** (same protein gel along with MAP2). Data are presented as mean ± SEM. **p < 0.01, ***p < 0.0001.

To explore the protective effect of VEN-02XX on neuronal populations in the 5XFAD/Rubicon KO model, key markers of neuronal integrity and health were evaluated. Neuroinflammation and IL-1β activity have been implicated in the neurotoxicity induced by Aβ and hyperphosphorylated tau and in feedforward mechanisms of pathology induced by inflammatory cytokines released by neurons in AD^58,59^. The presence of Aβ and pTau in the 5XFAD/Rubicon KO mice and abrogation of the phenotype by VEN-02XX treatment prompted the investigation of structural integrity in the hippocampus and cortex. Consistent with previous reports, the 5XFAD/Rubicon KO mice had significant reductions in neuronal numbers as measured by NeuN staining in both the cortex and hippocampus when compared to WT mice (Fig. 7A–D and Supplementary Fig. 6). Treatment with VEN-02XX after disease onset had a robust dose-dependent and statistically significant neuroprotective effect both in the cortex and hippocampus (Fig. 7A–D and Supplementary Fig. 6). In addition to improving overall neuronal numbers, VEN-02XX treatment significantly increased both CA3 and dentate gyrus (DG) thickness (Fig. 7E). To explore this neuroprotective effect further, immunoblot analysis was performed on cortical and hippocampal lysates and demonstrated consistent improvements in overall NeuN protein expression in both regions in animals treated with VEN-02XX compared to vehicle (Fig. 7F–I).

**Fig. 8:**
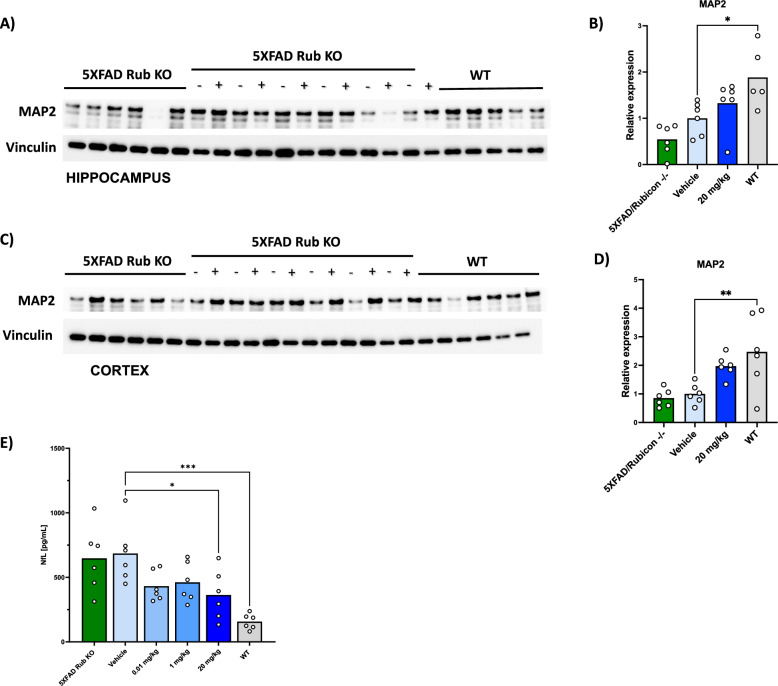
VEN-02XX treatment improves key neurostructural marker signatures consistent with neuroprotection. **A** Immunoblot analysis for MAP2 expression in hippocampal lysates; (−) animals refer to vehicle dosed 5XFAD/Rubicon KO mice and (+) animals refer to 20 mg/kg VEN-02XX dosed 5XFAD/Rubicon KO mice. **B** Quantification of band intensity for MAP2 normalized to vinculin for hippocampal samples (same protein gel along with NeuN). **C** Immunoblot analysis for MAP2 expression in cortical lysates; (−) animals refer to vehicle dosed 5XFAD/Rubicon KO mice and (+) animals refer to 20 mg/kg VEN-02XX dosed 5XFAD/Rubicon KO mice. **D** Quantification of band intensity for MAP2 normalized to vinculin for cortical samples (same protein gel along with NeuN). **E** Plasma NfL analysis (*n* = 6/group). Data are presented as mean ± SEM. *p < 0.05, **p < 0.01, ***p < 0.0001.

To evaluate dendritic integrity, we analyzed MAP2 expression in the cortical and hippocampal lysates. The expression of MAP2 was significantly reduced in the 5XFAD/Rubicon KO model compared to WT mice. When compared to vehicle treated mice, 20 mg/kg VEN-02XX treatment improved MAP2 levels in the 5xFAD/Rubicon KO mice trending towards healthy non-demented WT controls in both the cortex and hippocampus (Fig. 8A–D). The improvement in dendritic integrity demonstrated by MAP2 expression further suggests that NLRP3 inhibition with VEN-02XX is neuroprotective and supports restored neuronal function.

To further explore the possible mitigation of neuronal loss in the brain mediated by VEN-02XX treatment, we measured plasma levels of neurofilament light chain (NfL). NfL is a neuronal scaffolding protein important for maintaining the structural integrity of neurons. NfL is released upon axonal injury leading to increased concentrations in cerebrospinal fluid and blood. NfL has emerged as an important biomarker for several NDDs including AD^60^, as several reports have suggested that plasma NfL levels correlate with axonal degeneration and neuronal death in patients with AD. Importantly, NfL modulation by tofersen was the basis for its recent approval in ALS^61,62^ highlighting the clinical validity and significance of this biomarker. Consistent with a robust neurodegenerative phenotype, 5XFAD/Rubicon KO mice had significantly higher plasma NfL levels compared to WT animals (Fig. 8E). Despite a lack of a dose-response, plasma NfL levels were significantly reduced in the 5xFAD/Rubicon KO mice following VEN-02XX treatment at the higher dose (20 mg/kg).

When evaluated in concert with cortical and hippocampal immunohistochemistry, these data further support a neuroprotective role of NLRP3 inhibition using VEN-02XX in neurodegenerative indications including AD pathology.

## Discussion

Neuroinflammation, and the activation of the NLRP3 inflammasome, have been heavily implicated in the pathogenesis of multiple NDDs including AD. A host of activating stimuli including Aβ exist that promote and sustain NLRP3 activity leading to increased production of IL-1β and IL-18. Sustained inflammatory activation subsequently drives key aspects of AD pathology including both the accelerated deposition of Aβ and the hyper-phosphorylation and aggregation of tau^63,64^. More importantly, IL-1β supports the increased production of other pro-inflammatory cytokines in the NDD brain, including TNFα, IL-6, and others. Direct effects of these cytokines on neurons promote neuronal stress and ultimately activation of cell death mechanisms including pyroptosis, apoptosis and necroptosis. Emerging studies have revealed the robust nature of neuroinflammation and the NLRP3 inflammasome as putative therapeutic targets for the treatment of AD^2,3,9,14^. To date, most approaches have focused on germ-line genetic modulation or pharmacological inhibition using prophylactic dosing of non-CNS penetrant inhibitors of key pathway regulators including NLRP3 and caspase-1 amongst others^36,65–69^.

In the present study we evaluated for the first time the therapeutic efficacy of targeting the NLRP3 inflammasome in NDDs using a relevant murine model of AD, the 5XFAD/Rubicon KO mouse, which recapitulates the key features of human disease^29–33^. Until now, our understanding of the therapeutic benefit of blocking NLRP3 in AD is derived from several publish reports of pharmacologically blocking NLRP3 *before* the onset of disease symptomology and pathology in animal models of AD. Therefore, we set out to explore the *therapeutic* benefit of targeting NLRP3 in 4-month-old 5XFAD/Rubicon KO mice with established cognitive impairment (Supplementary Fig. 2) as well as confirmed AD pathology^32^. We found that blocking NLRP3 with our novel and brain-penetrant inhibitor VEN-02XX *after* the onset of disease symptomology is sufficient to promote several disease-modifying benefits in the 5XFAD/Rubicon KO model. Notably, a profound, dose-dependent and statistically significant improvement was observed in learning and memory as well as anhedonia behavior all of which demonstrate the robust symptomatic efficacy of VEN-02XX. From a mechanistic perspective, we demonstrated for the first time that NLRP3 inhibition is sufficient to shift the microglial cellular architecture from a hyperactivated ameboid state to a homeostatic ramified state with commensurate reductions in key pro-inflammatory cytokines and chemokine including IL-1β, TNFα, IL-6, and IP-10. This robust suppression in inflammation further limited tau phosphorylation in the model and provided appreciable neuroprotection both in the cortex and hippocampus.

A key finding that further delineates the mechanistic contribution of inflammasome activation and moreover the pharmacological inhibition of NLRP3 using a brain penetrant inhibitor (VEN-02XX) is the dose-dependent reduction in amyloid burden following suppression of IL-1β production. We observed clear statistically significant reductions in plaque size with trending reductions in plaque numbers (though not significant) supporting the potential of NLRP3 inhibition as putative disease modifying approach to reducing amyloid pathology. Together, these findings further represent a core therapeutic relevance of NLRP3 inhibition and a contextual mechanistic link between CASM-deficiency and amyloid pathology. To date, all approaches demonstrating that NLRP3 inhibition improves amyloid burden in AD have been performed using germline deletion of NLRP3 or caspase-1, and to our knowledge this is the first study to demonstrate a potential capacity for NLRP3 inhibition to reduce amyloid burden and/or morphology in the context of a therapeutic paradigm. Although we hold short of claiming an appreciable reduction in amyloid pathology across all measurements, the decreased plaque sizes and trend in reduction of plaque numbers, provides an optimistic basis that pharmacological NLRP3 inhibition as a therapeutic treatment may have a positive impact on amyloid pathology, even if not to the extent of germline NLRP3 deletion. Though beyond the scope of this study, it would be interesting to evaluate the effects of VEN-02XX and NLRP3 inhibition on amyloid pathology longitudinally over an extended dosing regimen to determine if statistically significant reductions could be achieved.

As described above, the 5XFAD mouse model and the 5XFAD/Rubicon KO model display varying degrees of tau phosphorylation and in certain instances aggregation typically between 6–8 months of age in the 5XFAD mouse and ~4 months for the 5XFAD/Rubicon KO model^56,57^. Considering this accelerated and enhanced tau phosphorylation in the model, we observed an appreciable reduction in tau phosphorylation at S396 upon VEN-02XX treatment, suggesting that neuroinflammatory signaling is likely driving or, at the very least, substantiating the tau pathology in the model.

We further hypothesize that the reduction in tau phosphorylation contributes to the neuroprotective effect of VEN-02XX treatment in addition to limiting cytokine and chemokine levels and thereby the direct exposure of neurons to death inducing stimuli. Although beyond the scope of the present study, to better characterize the mechanistic contribution of limiting tau phosphorylation in the 5XFAD/Rubicon KO model and the relevance to therapeutic engagement of NLRP3, additional studies evaluating other pTau isoforms, including S202/T205, S404, and others, which have been shown in the 5XFAD background previously^56,57^, need to be performed.

In addition to the changes observed in microglia, neuroinflammation, and tau phosphorylation, it was demonstrated by immunohistochemistry that a fundamental change in neurodegeneration was occurring in response to VEN-02XX treatment. Our evaluation of NfL, a clinically validated biomarker of neurodegeneration, revealed a significant signature of this biomarker in the plasma of 5XFAD/Rubicon KO mice, consistent with what is often observed in neurodegenerative diseases including in human AD patients (Quiroz et al.^70^). Importantly, we saw significant plasma NfL reduction at the highest VEN-02XX dose (20 mg/kg) in this model, with an absence of a dose-response likely attributable to the variability of this endpoint. Interestingly, this variability is similarly observed in human AD patients^71^. A larger study that is accurately powered for plasma biomarker analysis is likely needed to determine the measure of plasma NfL as an appropriate biomarker readout of the effect of NLRP3 inhibition using VEN-02XX and correlation to the improved behavior and neuropathology. Nevertheless, it is encouraging that disease associated changes were observed in the mouse model and moreover that trending improvements in that model were seen in response to NLRP3 inhibition.

In addition to plasma NfL, key markers of neuronal health in brain tissue from the 5XFAD/Rubicon KO model were measured and demonstrated lower expression of NeuN and MAP2 compared to WT counterparts, further suggesting an alteration in neuronal architecture and ultimately neurodegeneration. From a therapeutic perspective, although not statistically significant in the present study, VEN-02XX treatment improved both NeuN and MAP2 levels consistent with a neuroprotective efficacy. Extending the therapeutic duration of VEN-02XX beyond the 9-week timeframe would potentially allow for improvements in neuronal biomarker profiles while maintaining the robust symptomatic and disease-modifying efficacy observed in the study.

In the present study we were focused on evaluating intracellular markers of neurons and neuronal integrity including NeuN and MAP2. For future evaluation it may be informative to interrogate the consequences of NLRP3 inhibition on synapses and synaptic function. A potential confounding complexity with key synaptic markers including PSD95 however likely exists, especially in the 5XFAD/Rubicon KO model. Inhibition of the recycling endosome decreases PSD95 and GluA1levels and surface AMPA receptor levels at synapses^72^. Since the loss of single membrane LC-3 lipidation in the 5XFAD/Rubicon KO mouse suppresses the recycling of a variety of receptors through inhibition of CASM, it is plausible that impairment in recycling could similarly affect PSD95, AMPA receptor, and others. The role of CASM in regulating receptor recycling has best been characterized in microglia for receptors including TLR4, TREM2, and CD36 as well as in the heart with receptors including B1-adrenergic receptor^73^. Rubicon and single membrane lipidation of LC-3 also functions in neurons and recently has been shown to repress Aβ production^33^. The 5XFAD/Rubicon KO model features global deletion of Rubicon and therefore the loss of single membrane LC3-lipidation in neurons likely contributes to both the observed phenotype and changes in synaptic integrity and function, which has not been explored in detail.

As a transitory point, it is presumable that like NLRP3 signaling and neuroinflammation, Rubicon and single membrane LC3-lipidation likely function in the context of other NDDs in addition to AD. Emerging evidence evaluating neuroinflammatory signaling across broad NDDs supports the notion that shared mechanisms of regulation exist both in microglia and in neurons. In a strikingly similar manner to Aβ and tau in AD, other protein aggregates including α-synuclein in PD, and TDP-43 in ALS have been shown to activate microglia as well as trigger mitochondrial dysfunction and ROS production^74,75^. As potent activators of NLRP3 signaling, these data suggest that the NLRP3 inflammasome is a central regulatory hub for the establishment and propagation of pathology-associated reactive microgliosis. Targeted inhibition of NLRP3 may provide a robust approach for dampening or even reversing pathological microgliosis across neurodegenerative disorders or neuroinflammatory diseases. An appreciable amount of evidence supporting NLRP3 inhibition as a pan-therapy for neurodegeneration has emerged in recent years with other NLRP3 inhibitors. This provides fundamental validation in a wide range of NDDs including AD, PD, and ALS amongst others, even though these NLRP3 inhibitors lack either target specificity or have poor CNS-penetration properties. Studies performed herein using VEN-02XX as a specific NLRP3 inhibitor with robust CNS-penetrant properties further substantiate the potential of NLRP3 inhibition as a broad therapeutic mechanism of action against neuroinflammation and neurodegeneration.

In conclusion, the NLRP3 inflammasome represents one of the major therapeutic targets for NDDs including AD. Pervasive neuroinflammation can be referred to as the third “key hallmark” of AD pathology in addition to Aβ deposition and tau pathology. Using a murine model of AD that accurately mimics multiple facets of human neurodegenerative diseases, we evaluated the therapeutic capacity of targeting the NLRP3 inflammasome using a novel and highly selective brain penetrant inhibitor. Treatment using VEN-02XX after disease establishment had robust symptomatic effects, highlighted by a reversal in impaired cognition. Consistent with alleviating the primary symptom of AD, VEN-02XX demonstrated potential disease-modifying effects through reductions in neuroinflammation, tau pathology and potentially amyloid pathology, together, resulting in the reshaping of the microglial compartment towards a homeostatic state. Cumulatively, these findings validate inhibition of NLRP3 in the CNS as a viable therapeutic target in AD and establish the potential in other NDDs, including PD and ALS, and further elucidate the mechanistic contribution of CASM-deficiency and inflammatory priming in neurodegeneration.

## Methods

### Animals

5XFAD transgenic mice carrying five mutations: Swedish (K670N and M671L), Florida (I716V) and London (V717I) in human APP695 and human PS1 cDNA (M146L and L286V) under the transcriptional control of the neuron-specific Thy-1 promoter were purchased from The Jackson Laboratory. Rubicon−/− mice are available from The Jackson Laboratory. 5xFAD, Rubicon−/− were previously established and backcrossed >5 generations to establish consistent C57BL6/J background. SNP analysis was previously performed to ensure a background identity of greater than 97%. Unless otherwise noted, all experiments were performed on mixed sex cohorts at 4-months of age with littermate controls. All animal studies were approved by the University of South Florida Institutional Animal Care and Use Committee. All mice were housed in pathogen-free facilities, in a 12-h light/dark cycle in ventilated cages, with chow and water supply ad libitum.

### Dosing Schedule

5XFAD/Rubicon KO mice received vehicle (0.5% w/v methylcellulose/0.1% w/v Tween^®^ 80 in water) or VEN-02XX (0.01, 1, or 20 mg/kg) every day by oral gastric gavage for 9 weeks.

### Genotyping

To ensure proper genotypes, genomic DNA was isolated from tail snips at weaning. Standard PCR was used for the amplification of target sequences for each gene of interest and resolved on an agarose gel followed by visualization using SYBR Safe DNA staining. Imaging of the gels was performed on an Azure Biosciences UV Imager. Refer to Supplementary Table 1 for information on primers used.

### Blood and tissue collection

Blood was collected via submandibular puncture. Briefly, mice were immobilized, and a small puncture was made in the submandibular vein using a sterile 28 g lancet. Blood was collected using a capillary tube coated in 0.5 M EDTA into separation tubes for plasma as indicated. Blood was subsequently centrifuged for 15 min at 13,000 rpm in a refrigerated centrifuge. Plasma was harvested as indicated and was either used immediately or stored at −80 °C until use.

For post-mortem tissue collection, mice were anesthetized by isoflurane inhalation and perfused with 20 mL of sterile 1X PBS prior to tissue collection.

### Tissue sectioning and immunohistochemistry

Following post-mortem collection, the brain was placed in a mouse brain matrix and sliced along the sagittal suture to separate the hemispheres. The left hemisphere was immediately processed for downstream biochemical analyses by snap freezing in liquid nitrogen. The right half was drop-fixed in 4% PFA in PBS for 24 h and then washed 5x in fresh PBS before being submerged in 30% sucrose cryoprotectant solution for 24 h. After 24 h, brains were frozen in cryomolds in a 2:1 mixture of 30% sucrose and optimal cutting temperature compound (OCT), cryomolds were stored at −80 °C until use. For slide preparation, brains were allowed to acclimatize to −20 °C in a Thermo Shandon cryotome. After equilibration at −20 °C, tissue was cryostat sectioned at 25 μm (cortex) or 40 μm (free floating sections for hippocampus). Steam-mediated antigen retrieval was performed using 1x citrate buffer. Sections were rinsed in PBS, blocked in 3% bovine serum albumin/0.2% Triton X-100/PBS for 1 h, and incubated with primary antibodies in blocking buffer overnight at 4 °C (1:200 goat anti-Iba1 [Novus Biologicals, Centennial, CO], 1:500 mouse anti-Aβ 82E1 MOAB [Immuno-Biological Laboratories, Inc., Spring Lake Park, MN], 1:200 rabbit anti-pTau S396 [Cell Signaling, Danvers, MA], 1:200 mouse anti-NeuN [Cell Signaling]). Sections were incubated with appropriate secondary antibodies (Jackson ImmunoResearch, West Grove, PA) for 1 h at room temperature. For the complete list of antibodies please reference Supplementary Table 2. ProLong™ Diamond or ProLong™ Gold Antifade Mountant with or without 4’,6-diamidino-2-phenylindole (DAPI) counterstain (Thermo Fisher Scientific, Waltham, MA). Slides were imaged on a Zeiss Axio-observer A1 inverted microscope with an Andor iXon EMCCD camera and X-Cite LED fluorescence unit. Image analysis was performed using Fiji and 3DMorph AnalyzeSkeleton for microglial analyses.

### Behavior

#### Sucrose preference test

Basal preference to sucrose was first assessed on all enrolled mice. Individually housed mice were acclimated to the testing room for 48 h and to the two-bottle setup, where both bottles contained water, for 3 days. A 2% sucrose solution replaced the water in one bottle, and water consumption was measured for 4 consecutive days, while rotating the position of the sucrose-containing bottle. At the end of the testing period, both bottles were measured, and sucrose preference was calculated by dividing the intake from the sucrose bottle by the intake from both bottles, expressed as the percentage of sucrose preference. Preference to sucrose was re-assessed at 6- and 9-weeks following dosing with VEN-02XX. The test was performed as described above, with one bottle switched for 3% sucrose water. As before, bottles were rotated daily to reduce bias and leakage concerns, and the consumption was monitored for 4 days.

#### Spontaneous alternation

Spontaneous alternation was measured in a standard Y-maze setup. Mice were acclimated to the testing room for 48 h. In each 5-min trial, percent spontaneous alternation was measured as the number of entries in the three arms divided by the total number of alternations minus 2^32^. Any mouse with less than 5 arm entries during the 5 min trial was excluded from the analysis.

#### Novel object recognition

Mice were acclimated to the testing room for 48 h. For habituation, mice were allowed to explore an open-field box (50 cm × 50 cm) for 15 min per day over the course of 48 h. Mice were then exposed to two identical objects for 10 min on the day of testing. One hour later, a novel object was introduced, and mice were allowed to explore for 5 min during the test phase. The time spent exploring each object was quantified using AnyMaze video tracking software using the manufacturer define NOR protocol. Novel object preference (%) and the discrimination index (time with novel)/(novel + familiar) * 100) were calculated for each mouse using AnyMaze software^32^.

### Tissue homogenization and cytokine/chemokine quantification

Brain tissue was homogenized in PBS, pH 7.4 with 1X Halt™ protease inhibitor cocktail (Thermo Fisher Scientific, Waltham, MA) using a Bead Ruptor homogenizer (Omni International, Kennesaw, GA). Homogenates were clarified by centrifugation at 10,000 × *g* for 10 min. The crude homogenate was used for quantification of VEN-02XX levels, as described below. Total protein concentrations were quantified from clarified supernatants in a Pierce BCA protein assay (Thermo Fisher Scientific). Cytokine and chemokine levels were quantified using an electrochemiluminescence immunoassay (ECLIA) for IL-1β (total), TNFα, IL-6 and IP-10 (U-PLEX Custom Biomarker Group 1 (mouse) assay, Meso Scale Discovery, Rockville, MD) following the manufacturer’s instructions. Neurofilament light chain plasma levels were quantified using an enzyme-linked immunosorbent assay (ELISA) from Uman Diagnostics (CAT#: 20-8002) following the manufacturer’s instructions.

### Drug quantification

Concentrations of VEN-02XX were determined by a liquid chromatography with tandem mass spectrometry (LC-MS/MS) method. A Shimadzu UPLC system coupled with a SCIEX 4500 mass spectrometer (AB Sciex, Foster City, CA) was used for sample analysis. Data were acquired using multiple reactions monitoring (MRM) in positive ion electrospray mode with an operating source temperature of 400 °C. Two transitions were monitored for VEN-02XX and the more sensitive transition with less interference was used for quantification. Refer to the Supplementary Methods for the complete methodology.

### Immunoblotting

Ten ug of protein was separated by gel electrophoresis (one gel loaded with cortex samples and one gel loaded with hippocampus samples), on a 4–15% gradient Tris-glycine gel and transferred to PVDF membranes using the TransBlot Turbo semi-dry transfer system (Bio-Rad Laboratories, Hercules, CA). The membranes were blocked in Tris-buffered saline, 0.1% Tween 20 (1X TBST) with 5% milk for 1 h at room temperature followed by overnight incubation at 4 °C with primary antibodies (1:1000 rabbit anti-NeuN [EMD Millipore, Burlington, MA] and 1:10,000 rabbit anti-Vinculin [Cell Signaling, Boston, MA]) diluted in TBST with 1% milk. For the complete list of antibodies please reference Supplementary Table 2. The next day, membranes were washed three times with 1XTBST for 10 min before incubation with HRP-conjugated secondary antibody (1:500 goat anti-rabbit [Cedarlane Labs, Burlington, ON]) for 90 min at room temperature. Membranes were then washed three times with 1XTBST for 10 min and developed with Pierce ECL western blotting substrate (Thermo Fisher Scientific) and visualized on a ChemiDoc imaging system (Bio-Rad Laboratories). Membranes were gently stripped with Restore PLUS western blot stripping buffer for 10 min (Thermo Fisher Scientific) and reprobed, as described above, for MAP2 (1:1000 rabbit anti-MAP2 [Abcam, Cambridge, UK]).

### Mouse pharmacokinetic study

Male C57Bl/6 mice (weight 20–30 g, age 6–8 weeks old) were obtained from Charles River Laboratories Canada, and acclimated for 5 days before the study. Three animals were dosed by oral gavage during 7 days with 20 mg/kg of VEN-02XX suspended in 0.5% w/v methylcellulose/0.2% w/v Tween 80, at a dose volume of 10 mL/kg. Food and water were available ad libitum to all animals. On day 7, serial blood samples were collected from a tail snip at 0.25, 0.5, 1, 2, 4, 6, and 24 h post-dose, and 12 mL of blood was added to a micro tube racked system pre-filled with 36 mL of 10 mM sodium citrate buffer (final blood/buffer ratio 1:3, or dilution factor of 4). The plate was stored at −20 °C until LC-MS/MS analysis.

### Mouse brain pharmacokinetic study

Five groups of *n* = 3 male CD1 mice (weight 20–30 g, age 6–8 weeks old) were dosed by oral gavage with 20 mg/kg of VEN-02XX suspended in 0.5% w/v methylcellulose/0.1% w/v Tween 80, at a dose volume of 10 mL/kg. Food and water were available ad libitum to all animals. At a specific timepoint for each group (0.25, 1, 2, 4, and 8 h), 0.2 mL of blood was collected from the dorsal metatarsal vein and transferred into plastic micro centrifuge tubes with EDTA-K2 as anticoagulant, then animals were terminally anaesthetized with a rising concentration of CO2 gas for about 1 min, brains were collected and rinsed with cold PBS. Blood tubes were centrifuged at 4000 × *g* for 5 min at 4 °C to obtain plasma, and brain samples were homogenized in 3 volumes of PBS. Samples were frozen and stored at −75 ± 15 °C prior to analysis.

### LC-MS/MS quantification of VEN-02XX

Concentrations of VEN-02XX in plasma and brain were determined by a LC-MS/MS assay. The plasma samples were thawed at room temperature and 20 μL aliquot of each sample was added to a 1.2 mL deep 96-well plate containing 20 µL ACN. The standard curve was prepared by making different dilutions in ACN. A 20 µL standard solution at each concentration was added into a 96-deepwell plate, followed by addition of 20 µL blank plasma or brain homogenate. Then, 200 μL of an internal standard mixture (5 ng/mL Glyburide and Loperamide in ACN) was added to each sample. The plates were vortexed and centrifuged at 4000 rpm for 10 min at 4 °C; 70 μL of the supernatant was diluted with 140 μL of water, and 10 μL of the solution was injected onto an analytical column. A Shimadzu UPLC system coupled with a SCIEX 4500 mass spectrometer (AB Sciex, Foster City, CA) was used for sample analysis. The mobile phase A was 0.1% FA in water and mobile phase B was 0.1% FA in 1/5 IPA/ACN. The gradient started at 10% B, held for 0.2 min and increased to 95% B in 1.3 min, maintained at 95% B for 0.7 min, and then decreased to 10% B within 0.01 min. The total flow rate was 0.5 mL/min, and samples were injected onto an Acquity UPLC BEH C18 (2.1 mm × 5 cm, 2.3 u) analytical column with a total run time of 2.5 min. Data were acquired using multiple reactions monitoring (MRM) in positive ion electrospray mode with an operating source temperature of 400 °C. Two transitions were monitored for VEN-02XX and the more sensitive transition with less interference (397.1 –> 270.1) was used for quantification.

### PK analysis

Pharmacokinetic parameters were determined by non-compartmental analysis using WinNonLin software (Phoenix^TM^, version 8.3), and the following parameters were calculated from the plasma concentrations versus time data: *T*_1/2_, *C*_max_, *T*_max_, AUCinf, AUClast. When appropriate, the brain Kp was calculated as AUC_last_(brain)/AUC_last_(plasma). Free brain partition coefficient (Kpuu) was calculated using the free fraction in plasma and brain measured in a high throughput equilibrium dialysis assay.

### VEN-02XX on target in vitro assays

VEN-02XX was profiled in vitro to confirm specific binding to human NLRP3 as well as activity in various cellular assays.

#### Surface plasmon resonance (SPR)

The human NLRP3 protein was immobilized onto a nickel-NTA surface (Xantec NiHC1500M). Assay was run in 25 mM HEPES pH 7.5, 150 mM NaCl, 5 mM MgCl2, 0.5 mM TCEP, 0.5 mM ADP, 0.05% Tween-20 and 1% DMSO at 25 °C. Single-cycle method was used, and compound was injected over surface with a contact time of 120 s on and 600 s off by Biocore 8 K machine (Cytiva). A 5-points solvent correction curve was run pre and post run to correct for refractive index of DMSO. Data was then analyzed using the Biacore evaluation software to report binding kinetic parameters.

#### Mouse whole-blood in vitro assay

Blood was collected from CD1 female mice in lithium-Heparin tubes (Sarstedt, Numbrecht, Germany). Freshly collected blood was distributed in each well of a 96-well round-bottom cell culture plate (Corning Life Sciences, Tewksbury, MA). Blood was primed with 1 µg/ml Lipopolysaccharide (LPS) (*E. coli* O26:B6, Millipore, Burlington, MA) for 4.5 h, incubated at 37 °C with 5% CO2. Following LPS priming, blood was incubated with VEN-02XX pre-diluted in HBSS (Thermo Fisher Scientific, Waltham, MA) with 2% DMSO, for 30 min (top concentration 25 µM; 3-fold titration). Following drug incubation, blood was stimulated with 5 mM ATP (Millipore) for an additional 30 min at 37 °C with 5% CO_2_ under continuous shaking (450 RPM), after which conditioned media was collected for analysis of secreted IL-1β in an electrochemiluminescence immunoassay (Mesoscale Discovery).

#### PBMC in vitro assay

Cryopreserved human PBMCs (iXCells Biotechnologies, San Diego, CA) were rapidly thawed and incubated overnight at 37 °C with 5% CO_2_ in Complete Media: RPMI 1640 medium (ATCC modification, Thermo Fisher Scientific) supplemented with 10% Fetal bovine serum (FBS, Thermo Fisher Scientific) and 1% Penicillin-Streptomycin (Thermo Fisher Scientific). The next day, cells were centrifuged (300 × *g*, 5 min), resuspended in Assay Media (Complete Media without FBS) and plated at 33,333 cells/well in a 96-well cell culture plate. PBMCs were then primed with 100 ng/ml LPS (*E. coli* O26:B6) for 4 h. Following LPS priming, PBMCs were incubated with VEN-02XX pre-diluted in assay media with 0.8% DMSO, for 30 min (top concentration 100 µM; 4-fold titration). Following drug incubation, PBMCs were stimulated with 10 µM Nigericin (Millipore-Sigma, Burlington, MA) for an additional 60 min, after which conditioned media was collected for analysis of secreted IL-1β in an electrochemiluminescence immunoassay (Mesoscale Discovery).

#### iPSC-derived microglia in vitro assay

*i*PSC-derived microglia (BX-0900, BrainXell, Madison, WI) were thawed and recovered as per the supplier’s instructions. Briefly, viable cells were counted post-thaw using Trypan Blue, 0.4% (Thermo Fisher Scientific) and 25,000 cells were seeded on poly-d-lysine coated 96-well plates in DMEM-F/12 medium (Thermo Fisher Scientific) supplemented with 1X B-27 supplement (Thermo Fisher Scientific), 1X N-2 supplement (Thermo Fisher Scientific), 1X Non-essential amino acids (Thermo Fisher Scientific), 1X Chemically defined lipid mix (Thermo Fisher Scientific), 1 mM GlutaMax (Thermo Fisher Scientific), 0.2 mM Ascorbic Acid (Millipore), 100 ng/mL IL-34 (PeproTech, Cranbury, NJ), 2 ng/mL TGF-β1 (PeproTech) and 20 ng/mL M-CSF (PeproTech). Fresh media was added 3 days after seeding, half of which was replenished with fresh media 6 days following seeding. The assay was performed 7 days after cells were seeded in 96-well plates. Two assay protocol variations were performed: cells were either pre-treated with VEN-02XX to block NLRP3 inflammasome formation and then primed with LPS (Fig. 1, D. Compound added before LPS priming) or were primed with LPS and then treated with VEN-02XX (Fig. 1, D. Compound added after LPS priming). For compound treatment, cells were treated with VEN-02XX for 1 h (top concentration 100 µM; 3-fold titration) to block NLRP3 inflammasome formation. For LPS priming, cells were primed with 100 ng/mL LPS (E.coli O26:B6, Millipore) for 4 h. At the end of either protocol, cells were stimulated with 10 uM Nigericin (Millipore) for 1 h, after which conditioned media was collected for analysis of secreted IL-1β, IL-18 and TNFα in an electrochemiluminescence immunoassay (Mesoscale Discovery).

### VEN-02XX in vitro selectivity assays

#### AIM2 in vitro assay

THP1-KO-NLRP3 cells (Invivogen, San Diego, CA), resuspended in RPMI 1640 (Thermo Fisher Scientific) supplemented with 25 mM HEPES (Thermo Fisher Scientific), 10% FBS (Thermo Fisher Scientific) and 1% Penicillin-Streptomycin (Thermo Fisher Scientific), were seeded at 75,000 cells/well in a 96-well cell culture plate and treated with 50 ng/ml Phorbol 12-myristate 13-acetate (PMA, Millipore) for 24 h at 37 °C with 5% CO_2_. The next day, the media was replaced with fresh media containing 2.2% FBS, and cells were pretreated for 60 min with VEN-02XX (top concentration 50 µM; 5-fold titration) before DNA transfection. Lipofectamine 2000 (Thermo Fisher Scientific), for a final concentration of 2 µg/ml, and Poly(dA:dT) (Invivogen), for a final concentration of 1 µg/ml, were mixed together in OptiMEM (Thermo Fisher Scientific) 5 min before their addition to the cells. After a 24-h incubation at 37 °C with 5% CO_2_, supernatants were collected and IL-1β levels determined in an electrochemiluminescence immunoassay (Mesoscale Discovery).

#### NLRC4 in vitro assay

THP1-Null2 cells (Invivogen), resuspended in RPMI 1640 (Thermo Fisher Scientific) supplemented with 25 mM HEPES (Thermo Fisher Scientific), 10% FBS (Thermo Fisher Scientific) and 1% Penicillin-Streptomycin (Thermo Fisher Scientific), were seeded at 65,000 cells/well in a 96-well cell culture plate and treated with 50 ng/ml PMA (Millipore) for 24 h at 37 °C with 5% CO_2_. The next day, the media was replaced with fresh media without FBS, and cells were pretreated for 60 min with VEN-02XX (top concentration 50 µM; 5-fold titration). During the incubation, the Needle-Tox solution was prepared by mixing LFn-Needle (expressed in-house) and *Bacillus anthracis* protective antigen (List Labs, Campbell, CA) to obtain final concentrations of 50 ng/ml and 100 ng/ml, respectively. Following the addition of Needle-Tox, cells were incubated for 5 h at 37 °C with 5% CO_2_. Supernatants were then collected and IL-1β levels were determined in an electrochemiluminescence immunoassay (Mesoscale Discovery).

#### NLRP1 in vitro assay

Normal Human Epidermal Keratinocytes pooled from juvenile donors (PromoCell, Heidelberg, Germany) were cultured in Keratinocyte Growth Medium 2 (PromoCell). Cells were seeded at 12,000 cells/well in a 96-well cell culture plate and incubated for 16 h at 37 °C with 5% CO_2_. The next day, the media was replaced with fresh media, and cells were pre-incubated for 60 min with VEN-02XX (top concentration 50 µM; 5-fold titration) before the addition of Val-boroPro mesylate (MedChemExpress, Monmouth Junction, NJ) at a final concentration of 7.5 µM. After a 30-h incubation at 37 °C with 5% CO_2_, supernatants were collected and IL-1β levels were determined in an electrochemiluminescence immunoassay (Mesoscale Discovery).

### Statistics

Cytokine levels, western blots, immunohistochemical analysis and the behavior data were analyzed using a One-way ANOVA with the Dunnett’s post hoc test comparing the several doses of VEN-02XX treated mice to vehicle controls using GraphPad Prism (v10). A ROUT outlier analysis was performed for each analyzed cytokine (*Q* = 1.0%), omitting one outlier mouse from the vehicle-treated group and one outlier mouse from the WT group. An unpaired *t*-test was performed to compare baseline sucrose preference at enrollment between WT and 5XFAD/Rubicon knockout mice.

## Supplementary information

Supplementary information

## Data Availability

The datasets used and/or analyzed in the current study are included in the manuscript or are available from the corresponding author on reasonable request.

## Abbreviations

AD: Alzheimer’s disease
Aβ: Amyloid beta
NLRP3: NOD-, LRR- and pyrin domain containing protein 3
IL-1β: Interleukin 1 beta
ROS: Reactive oxygen species
ASC: Apoptosis-associated speck-like protein containing a caspase recruitment domain
PAMP: Pathogen associated molecular pattern
DAMP: Danger associated molecular pattern
5XFAD: 5X familial Alzheimer’s disease
PBMC: Peripheral blood mononuclear cells
iPSC: Induced pluripotent stem cells
SPT: Sucrose preference test
NOR: Novel object recognition
CNS: Central nervous system
DAM: Disease associated microglia
IL-6: Interleukin 6
TNFα: Tumor necrosis factor-alpha
IP-10: Interferon gamma inducible protein-10
Iba1: Ionized calcium-binding adapter molecule 1
pTau: Phosphorylated tau
NeuN: Neuronal nuclei
MAP2: Microtubule-associated protein 2
DAPI: 4’,6-diamidino-2-phenylindole (DAPI)
NF-L: Neurofilament light
APP: Amyloid precursor protein
PS1: Presenilin-1
LC3: Microtubule-associated protein light chain 3
LANDO: LC3-associated endocytosis
TLR4: Toll like receptor 4
TREM2: Triggering receptor expressed on myeloid cells 2
CD36: Cluster of differentiation 36
