# Supplementary material for: Post-symptomatic NLRP3 inhibition rescues cognitive impairment and mitigates amyloid and tau driven neurodegeneration
Source: NPJ Dement. 2025 May 6;1(1):3. doi: 10.1038/s44400-025-00011-5 (PMC12055592; doi:10.1038/s44400-025-00011-5)

SUPPLEMENTARY FIGURES

for

Post-symptomatic NLRP3 inhibition rescues cognitive impairment and mitigates amyloid and tau driven neurodegeneration

AUTHORS

Anick Auger[a], Rania Faidi[a], Alexis D. Rickman[c], Carolina Pena Martinez[c], Austin Fajfer[c], Jeremy Carling[c] Addison Hilyard [c], Mubashshir Ali[c], Ryosuke Ono[c], Connor Cleveland[c], Ria Seliniotakis[a], Nhi Truong[c], Amandine Chefson[a], Marianne Raymond[a], Marie-Anne Germain[a], Michael A. Crackower[b], and Bradlee L. Heckmann[c*]

Supplementary Figure 1

A)

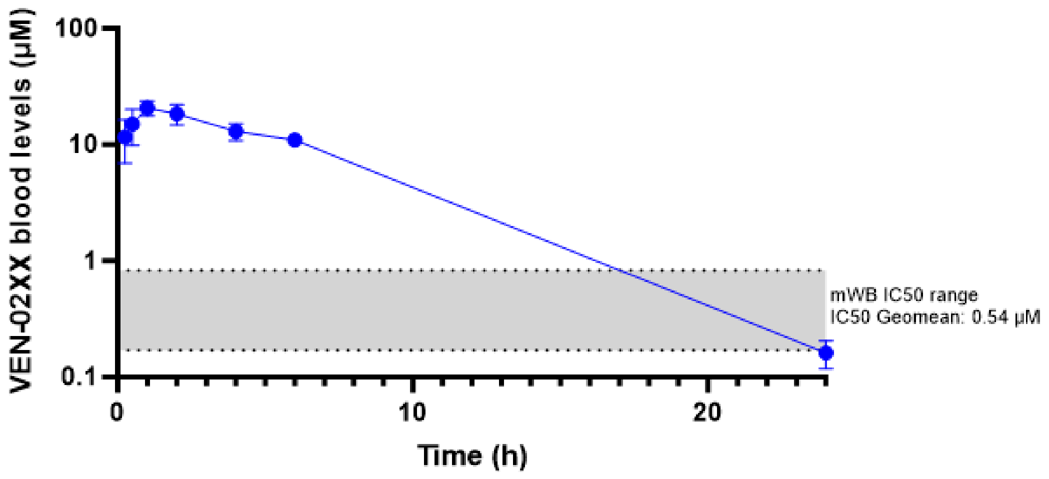

B)

	VEN-02XX Pharmacokinetic parameters
$T_{1/2}$ (h)	3.16
Tmax (h)	1.00
Cmax (µM)	20.70
AUC_{inf} (h*µM)	142.68

Supplementary Figure 1. Pharmacokinetic properties of VEN-02XX in WT mice.

A) Pharmacokinetic profile of VEN-02XX in blood following oral dosing (PO) with VEN-02XX at 20 mg/kg administered once daily (QD) for seven days in wild type (WT) mice. B) Pharmacokinetic parameters of VEN-02XX following PO dosing at 20 mg/kg (QD) for seven days.

Supplementary Figure 2

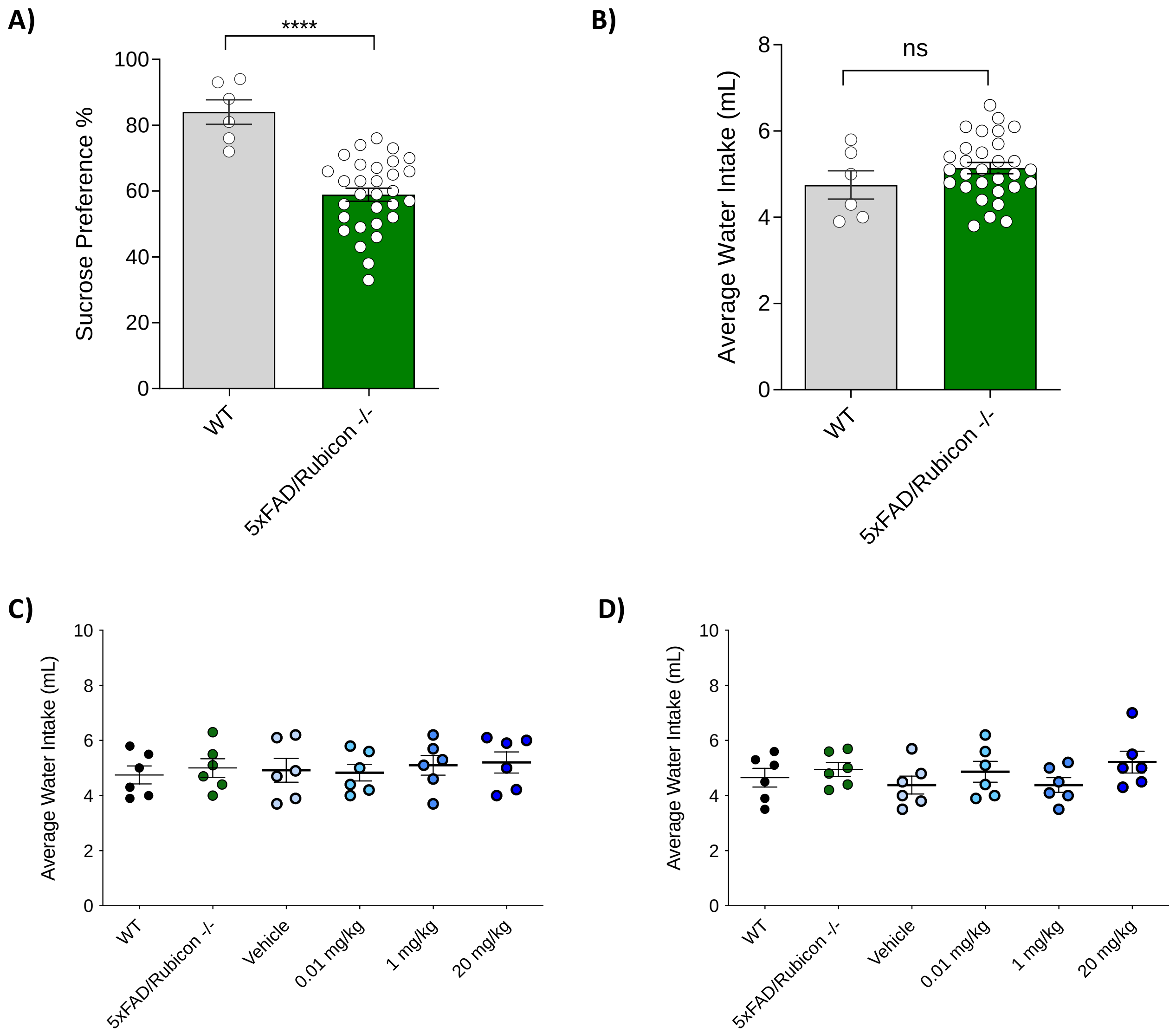

Supplementary Figure 2. Extended data for sucrose preference tests.

A) Pre-treatment sucrose preference to confirm cognitive deficits in study cohort animals. B) Water intake measurement for pre-treatment sucrose preference. C,D) Water intake measurement for 6-week and 9-week post-dose initiation sucrose preference tests, see Figure 2C and 2D. Data are presented as mean ± SEM. ****$p < 0.00001$.

Supplementary Figure 3

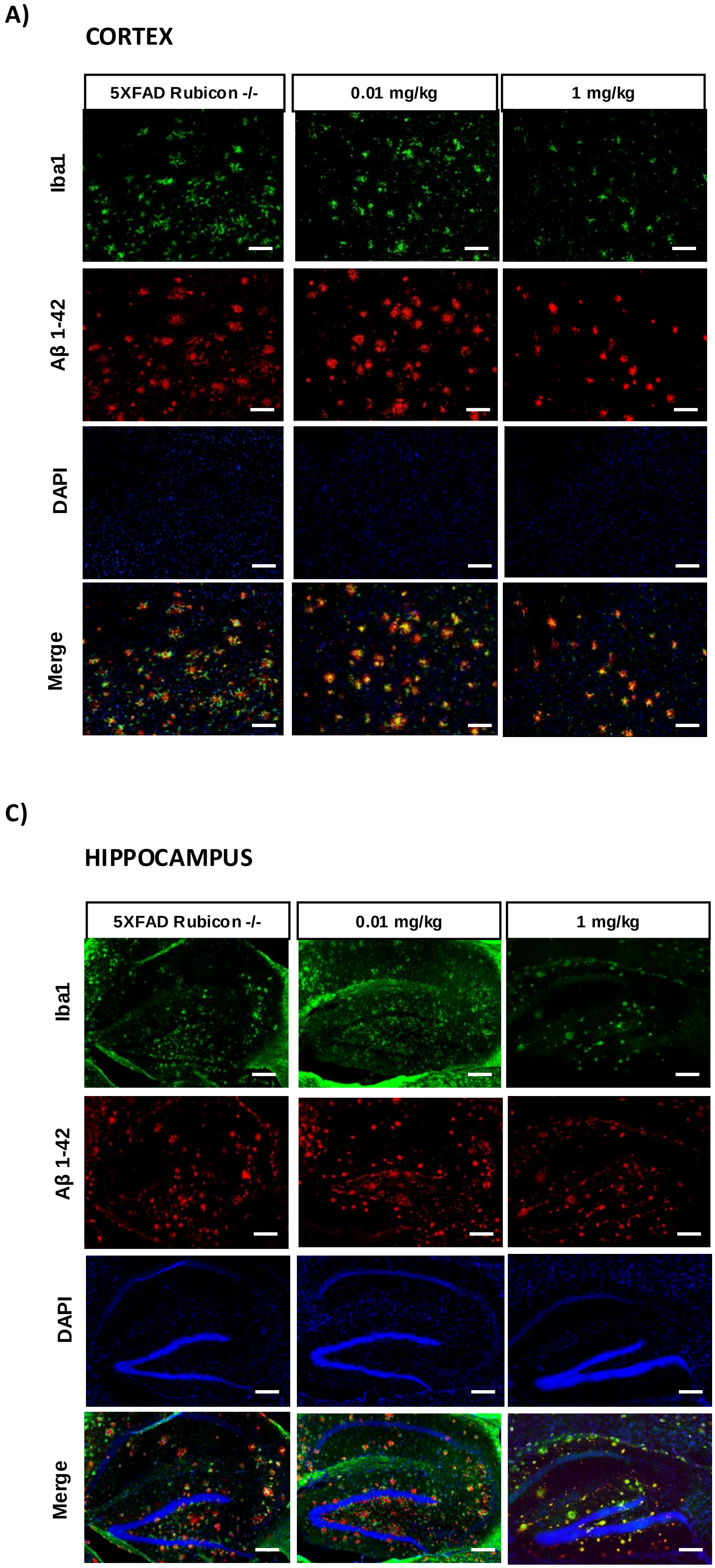

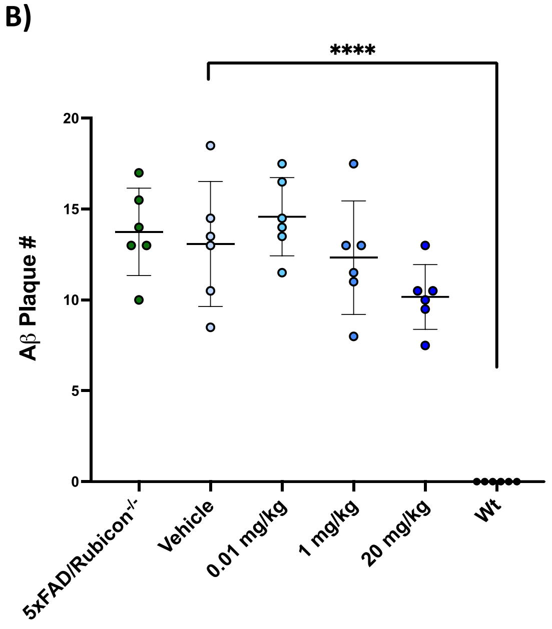

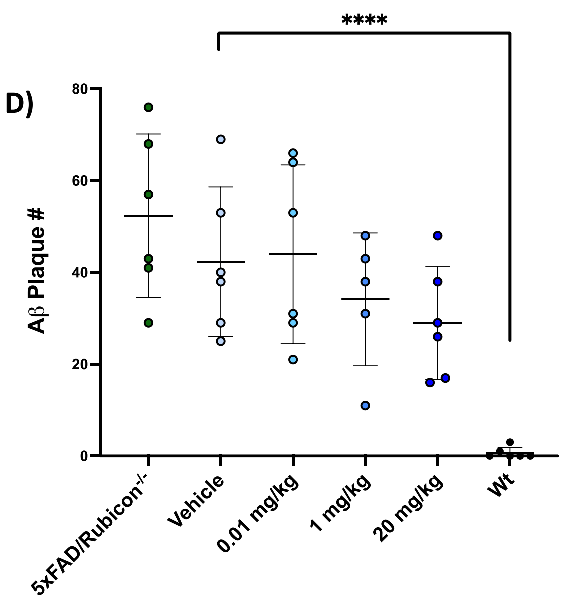

Supplementary Figure 3. Extended data for microglial activation state via Iba1 and amyloid pathology.

A) Representative imaging for cortex Iba1 (green), Aβ (red), and DAPI (blue) on additional dosing groups. Scale bars, 50um. B) Quantification of amyloid plaque # in cortex. C) Representative imaging for hippocampal Iba1 (green), Aβ (red), and DAPI (blue) on additional dosing groups. Scale bars, 100 um. D) Quantification of amyloid plaque # in hippocampus. See Figure 5 for additional quantification of plaque area.

Supplementary Figure 4

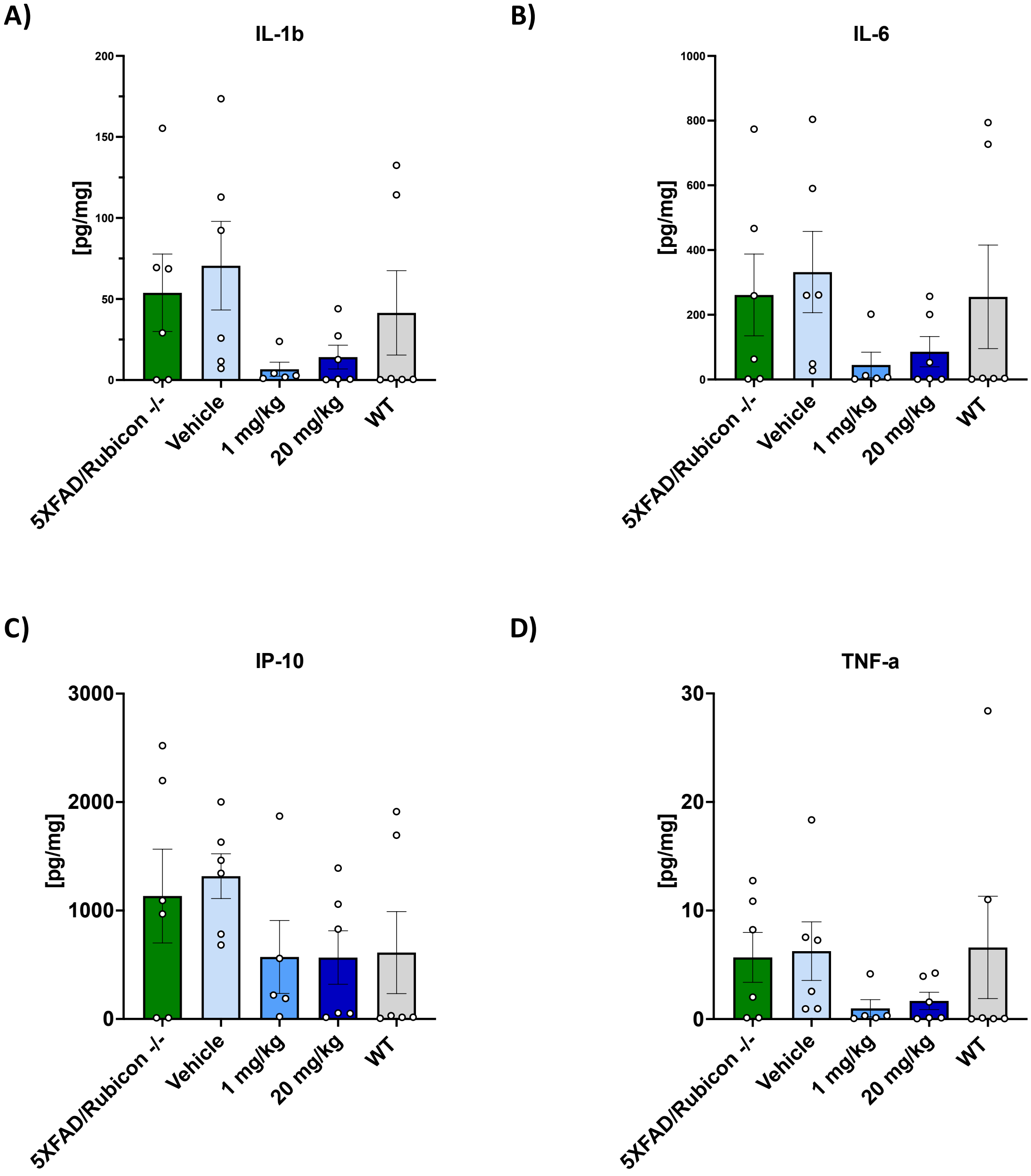

Supplementary Figure 4. Extended data for cortical cytokine levels in VEN-02XX treated mice.

A-D) Quantification (pg/mL) of cortical IL-1β (total), IL-6, IP-10, and TNFα in 5XFAD Rubicon KO mice receiving no treatment (green bars; n=6), vehicle (light blue bars; n=5), and 1 mg/kg (medium blue bars; n=6) or 20 mg/kg VEN-02XX (dark blue bars; n=6). Wild type (WT) mice receiving no treatment (n=5) are shown in gray bars. Data are presented as mean ± SEM.

Supplementary Figure 5

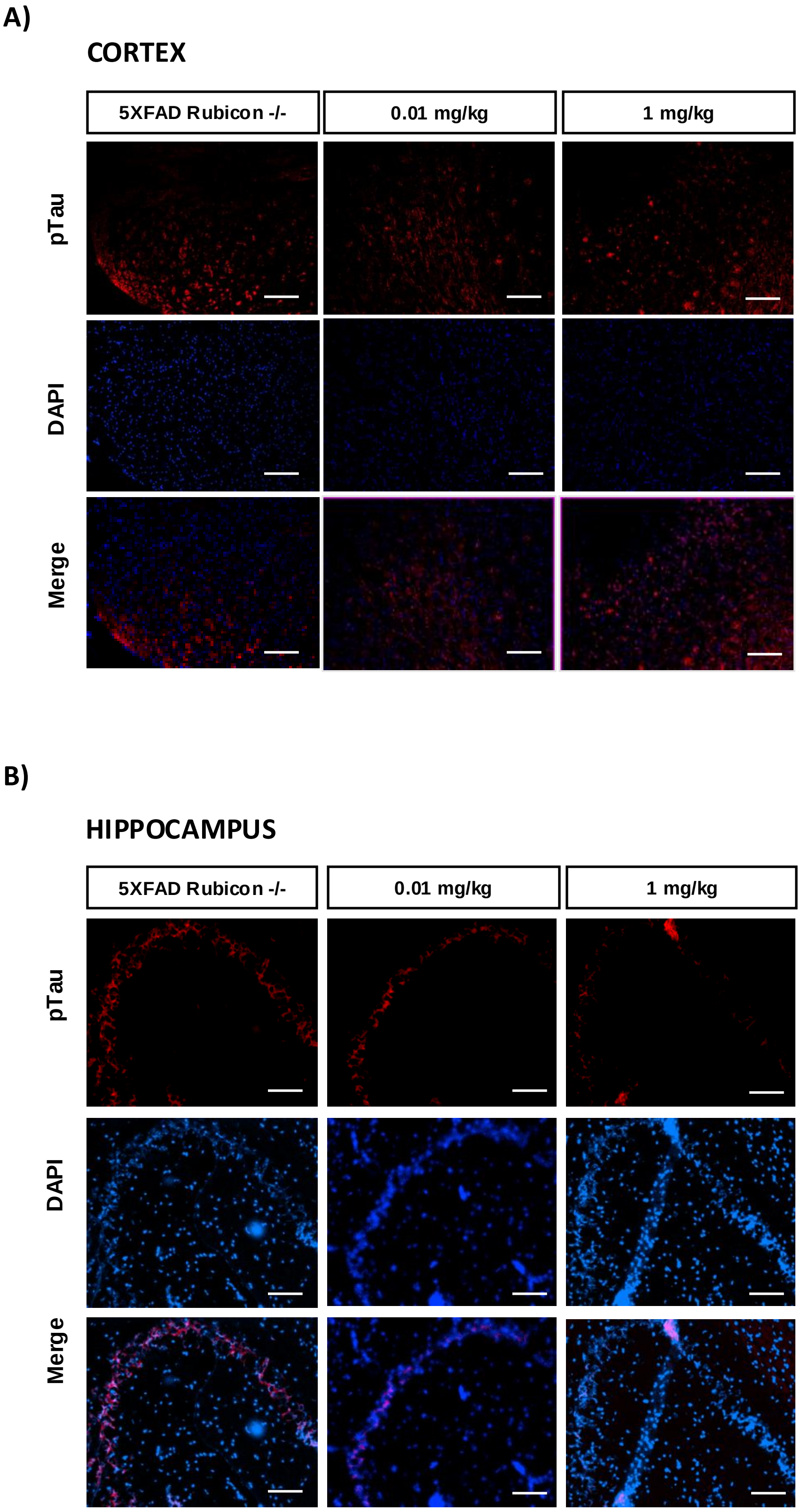

Supplementary Figure 5. Extended data for pTau analysis following VEN-02XX treatment.

A,B) Representative imaging for cortex and hippocampal pTau S396 (red) and DAPI (blue) on additional dosing groups. Scale bars, 50um for A, 100um for B. See Figure 6B and 6D for quantification.

Supplementary Figure 6

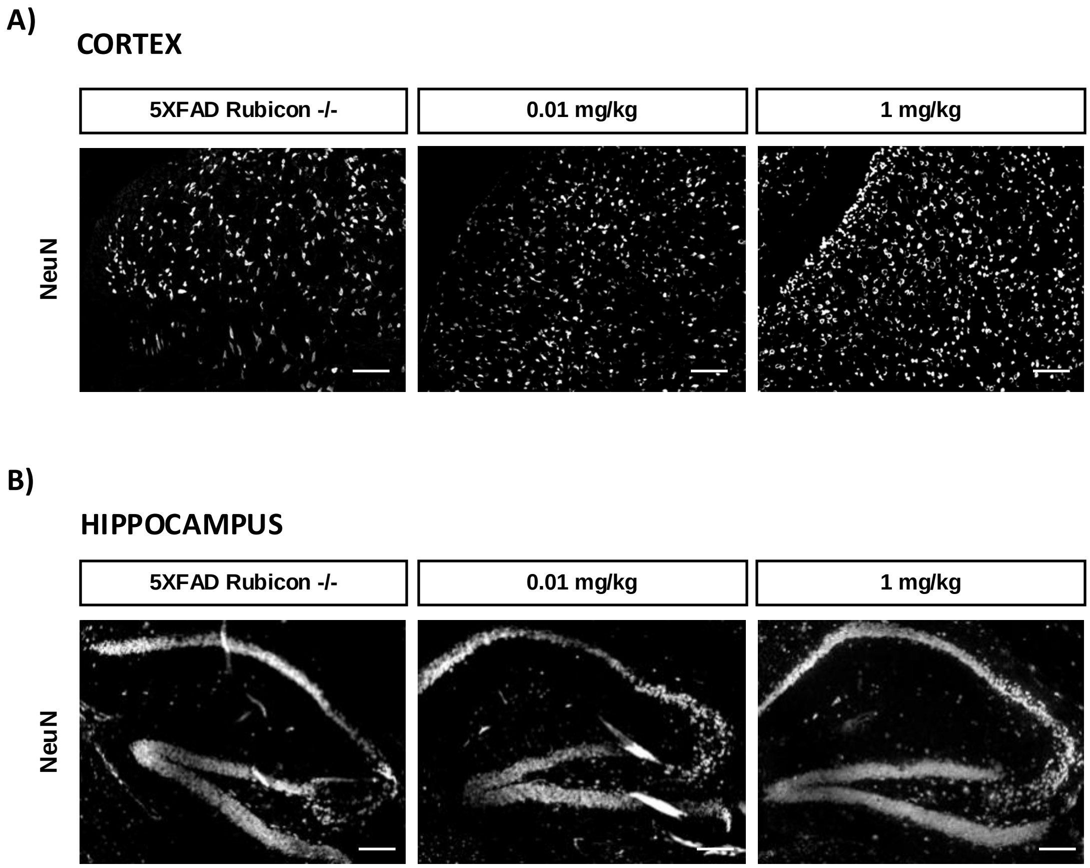

Supplementary Figure 6. Extended data for neuronal analysis following VEN-02XX treatment.

A,B) Representative imaging for cortex and hippocampal NeuN staining (monochromatic) on additional dosing groups. Scale bars, 100um. See Figure 7B,D,E for quantification.

Supplementary tables

Table 1. Primers for genotyping 5XFAD Rubicon KO mice

	Primer	Sequence
5XFAD	Common Forward	5’ – ACCCCCATGTCAGAGTTCCT – 3’
	Mutant reverse	5’ – CGGGCCTCTTCGCTATTAC – 3’
	Wildtype reverse	5’ – TATACAACCTTGGGGGATGG – 3’
Rubicon	Forward	5’ – AGCAGAGGGTTTTATGCGCT – 3’
	Reverse	5’ – CCACCACACCCAACTCTTCA – 3’

Table 2. Antibody Catalog List

	Supplier	Catalogue Number	Application	Dilution
NeuN	EMD Millipore	ABN78	Western blot	1:1,000
MAP2	Abcam	ab32454	Western blot	1:1,000
Vinculin	Cell Signalling	13901S	Western blot	1:10,000
Rabbit IgG horseradish peroxidase-conjugated	R&D systems	HAF008	Western blot	1: 5000
Aβ 82E1 MOAB	IBL	10323	IHC	1:500
PTau S396	Cell Signaling	9632	IHC	1:200
Iba1	Novus	NB100-1028	IHC	1:200
NeuN	Cell Signaling	24307	IHC	1:200

Supplementary Raw Immunoblots

- Gel and membrane stains corresponding to immunoblots in figures 7 & 8.

CORTEX

4-15% Tris Glycine stain free Gel #1

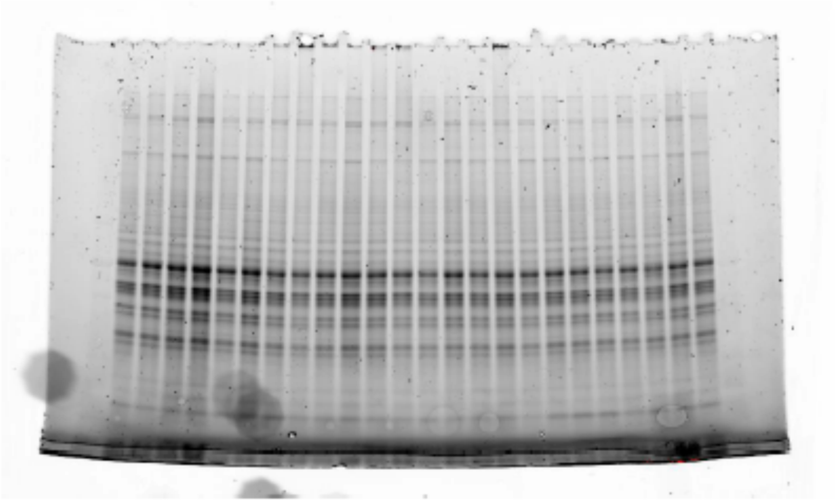

PVDF stain free membrane #1

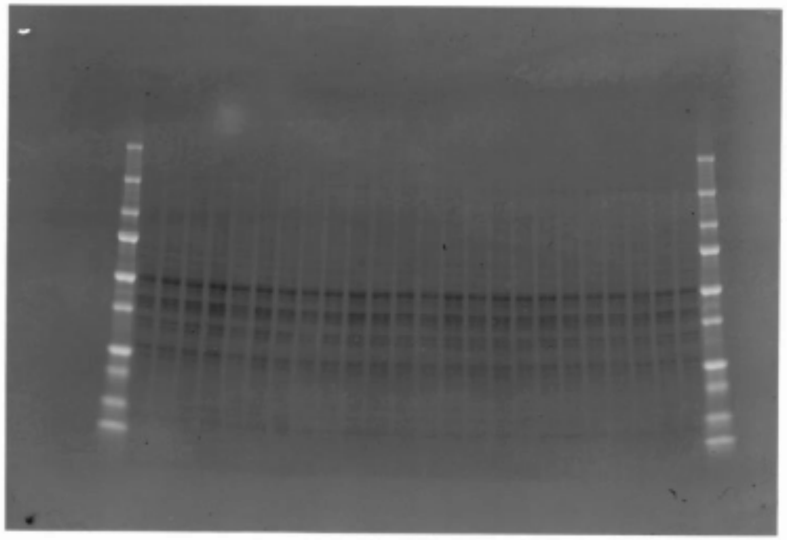

Figure 7G & 8C Cortex Loading Control

Vinculin – 124 kDa

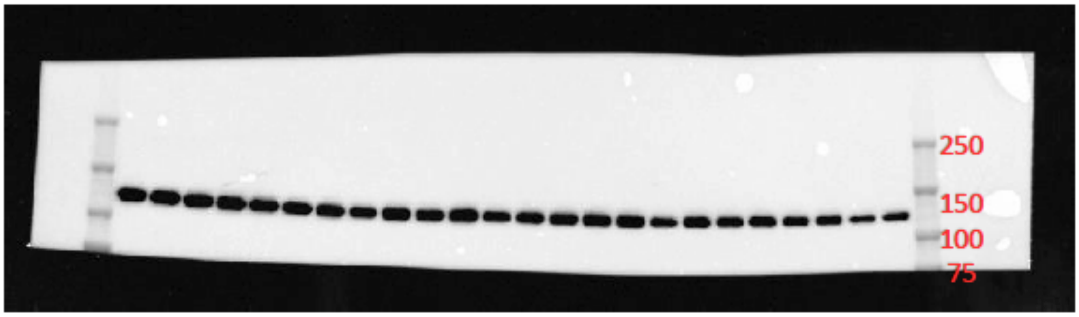

Figure 7G NeuN

NeuN – 48 kDa

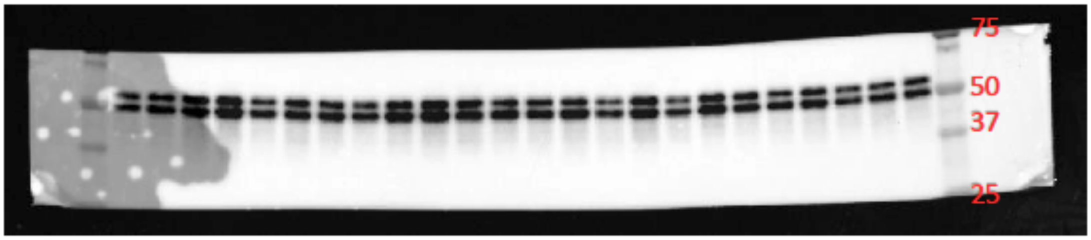

Figure 8C MAP2

MAP2 ~280 kDa

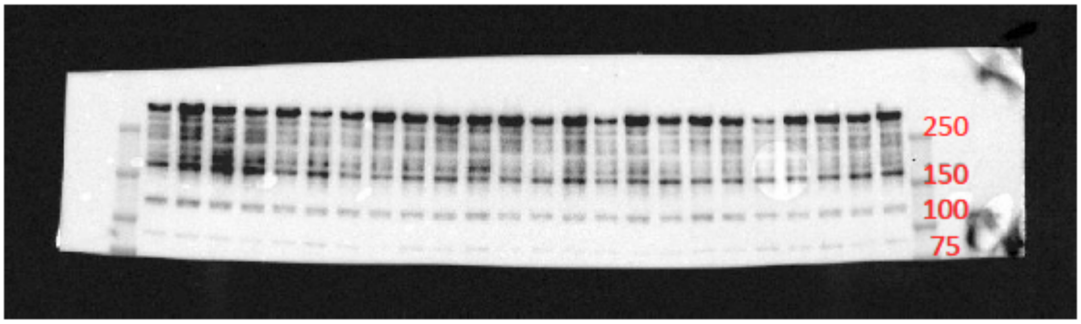

HIPPOCAMPUS

4-15% Tris Glycine stain free Gel #1

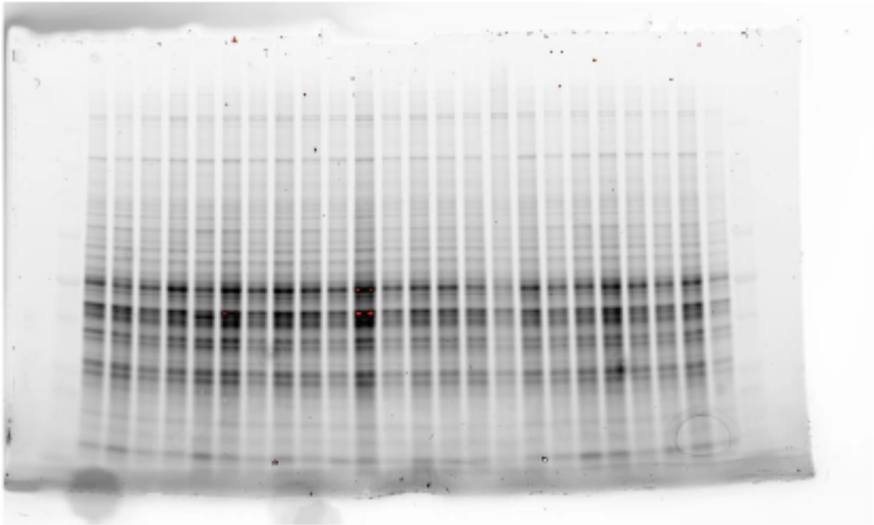

PVDF stain free membrane #1

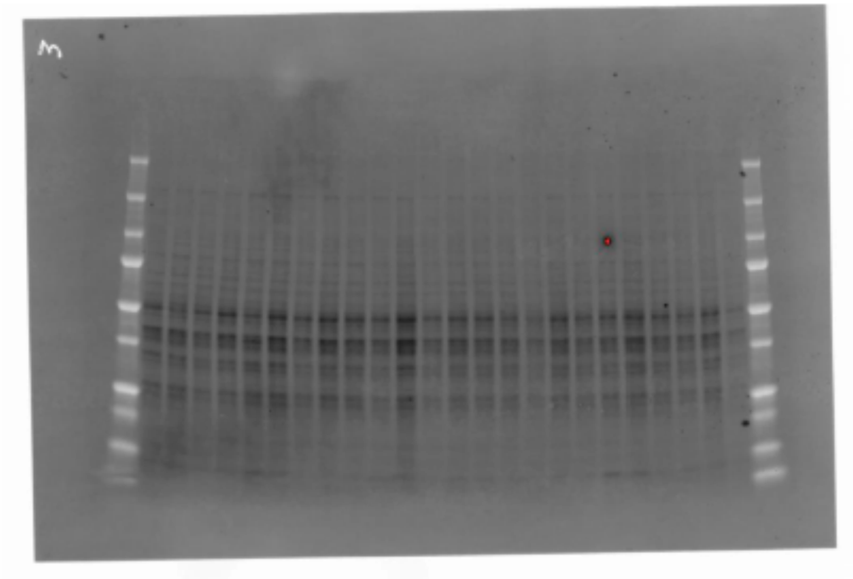

Figure 7F & 8A Cortex Loading Control

Vinculin – 124 kDa

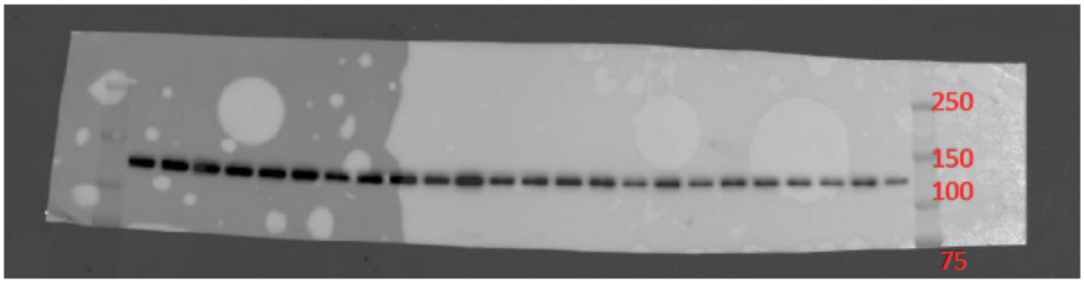

Figure 7F NeuN

NeuN – 48 kDa

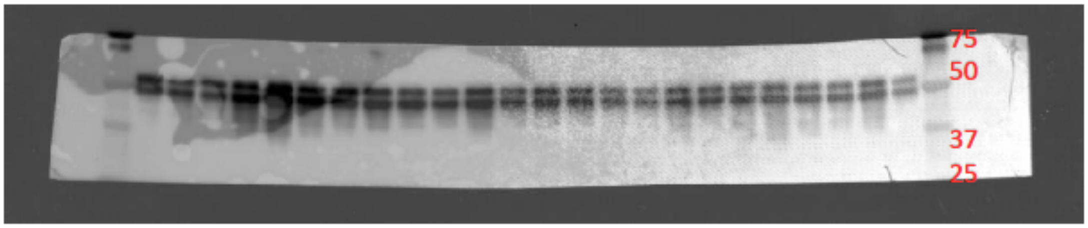

Figure 8A MAP2

MAP2 ~280 kDa

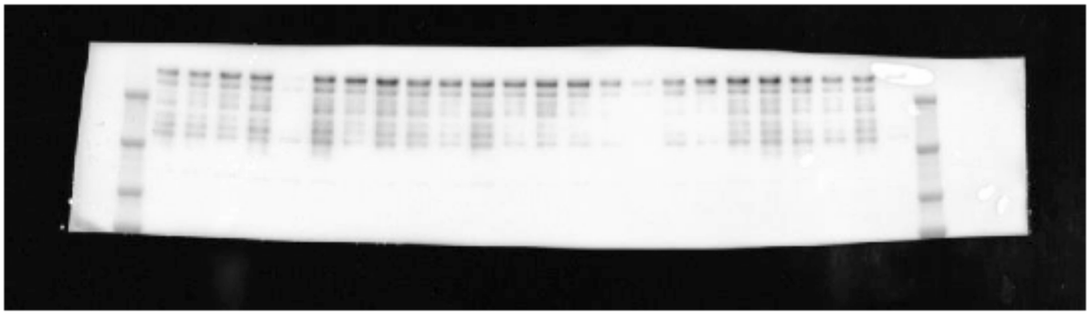

Supplement: Supplementary file 1 — Supplementary information [file 44400_2025_11_MOESM1_ESM.pdf]
